# A Latin American, Portuguese and Spanish consensus on a core communication curriculum for undergraduate medical education

**DOI:** 10.1186/s12909-016-0610-8

**Published:** 2016-03-28

**Authors:** Cristina García de Leonardo, Roger Ruiz-Moral, Fernando Caballero, Afonso Cavaco, Philippa Moore, Lila Paula Dupuy, Antonio Pithon-Cyrino, Mª Teresa Cortés, Marilen Gorostegui, Elizabete Loureiro, Josep Mª Bosch Fontcuberta, Luis Casasbuenas Duarte, Lara Kretzer, Emilia Arrighi, Albert Jovell, on behalf of the participants in the Consensus Panel

**Affiliations:** Escuela de Medicina, Universidad Francisco de Vitoria, Facultad de Medicina, Edificio E, Ctra Pozuelo-Majadahonda, Km 1,800, Pozuelo de Alarcón, Madrid 28223 Spain; Universidade de Lisboa, Faculdade de Farmácia, Lisbon, Portugal; P. Universidad Católica de Chile, Facultad de Medicina, Santiago de Chile, Chile; Universidad Maimonides, Facultad de Medicina, Buenos Aires, Argentina; Universidade Estadual Paulista, Faculdadede Medicina, Botucatu, Brazil; Universidad Nacional Autónoma de México, Facultad de Medicina, Ciudad de México, México; Universidad de Chile, Facultad de Medicina, Santiago de Chile, Chile; Universidade do Porto, Faculdade de Medicina, Porto, Portugal; Universidad Autónoma de Barcelona, Facultad de Medicina, Barcelona, Spain; Universidad de Antioquia, Facultad de Medicina, Medellín, Colombia; Universidade Federal de Santa Catarina, Faculdadede Medicina, Florianópolis, Brazil; Universidad Internacional de Cataluña, Barcelona, Spain; Foro Español de Pacientes (Spanish Patient Forum), Barcelona, Spain

**Keywords:** Physician patient communication, Interpersonal skills, Health communication, Delphi technique, Consensus method, Medical education, Medical curriculum, Learning outcomes

## Abstract

**Background:**

To present learning outcomes in clinical communication for a Core Curriculum for medical undergraduate students in Latin America, Portugal and Spain (LAPS-CCC) and to establish an expert network to support a transnational implementation.

**Methods:**

Through an iterative process, an international group of 15 experts developed an initial set of learning outcomes following a review and discussion of relevant international and local literature. A two-round Delphi survey involving 46 experts from 8 countries was performed. Quantative and qualitative analisis permited the definition of the final consensus.

**Results:**

The initial proposal included 157 learning outcomes. The Delphi process generated 734 comments and involved the modification, deletion and addition of some outcomes. At the end of the process, a consensus was reached on 136 learning outcomes grouped under 6 competency domains with a high overall acceptance (95.1 %).

**Conclusions:**

The learning outcomes of this proposal provide a guide to introduce, support and develop communication curriculae for undergraduate medical studies in the countries involved or in other Spanish- or Portuguese-speaking countries.

**Electronic supplementary material:**

The online version of this article (doi:10.1186/s12909-016-0610-8) contains supplementary material, which is available to authorized users.

## Background

In healthcare, communication between health providers and patients and their families has become a core competency for achieving quality objectives [1]. Research has shown positive associations not only between physician-patient communication and important healthcare outcomes [2–4], but also between specific educational strategies and the acquisition and application of communication skills by students and health providers [5–7].

There are a number of documents defining good practice in physician-patient relationship and that can be useful for planning and developing training programmes and appropriate assessment strategies in this area [8–11]. In addition, several interesting and well-structured proposals have been published in Canada, UK, Germany and Europe [12–15]. These proposals have been reached by consensus and are intended to be reference-guides for the learning outcomes (LO) on communication that should be included in the undergraduate curricula for medicine and other health professions.

In Latin America, Portugal and Spain (LAPS), medical schools are in the process of integrating physician-patient communication skills into their under-graduate curricula; some medical schools have proposed general regulatory guidelines with this aim [16–20]. However, few medical schools that have formally introduced communication skills into their curricula and there is a great diversity not only in the type or content of competencies required, but also in terms of when, where and how communication skills should be taught [21–27]. As there is significant exchange of health providers between these countries [28], there is a strong argument to implement initiatives similar to that of the Bologna Declaration [29] which recommends that an approximation based on common and well-defined competencies should be incorporated into medical schools in order to facilitate comparability and to allow exchange between schools. No consensus statements about LO on communication exists for Spanish- or Portuguese-speaking countries. The authors considered it appropriate to develop their own consensus rather than proposing partial or total application of the LO taken directly from other consensuses for the following reasons: (i) There is a need to assess whether the proposals from other consensus statements apply to the current existing cultural/social and educational context in LAPS, (ii) A consensus statement will facilitate the introduction and development of training in comunicaction skills in LAPS medical schools and may promote the standardisation of LO between the educational institutions of these countries, (iii) The process of creating a consensus statement about communication LO would mobilise a group of skilled and influential people working in the area of communication in health; the creation of such a network may be able to facilitate the introduction and development of training in communication skills for health professionals in Spanish- and Portuguese-speaking countries.

The main objective of this study was to reach a consensus among experts from LAPS to define LO in patient-physician communication that medical students should be acquired during their undergraduate studies (LAPS-CCC). This consensus aims to provide guidance and encouragement for implementing and developing communication skills in the curricula of those medical schools that have not incorporated them yet, and to support faculty and curriculum planners in their efforts in this task. The definition of the consensus contents as expected LO provides a basis for applying valid methods for teaching and assessment throughout the curriculum.

## Methods

In brief, a Scientific Committee made up of 15 members which were established ad hoc, undertook a review of the literature on the subject. They exchanged relevant information on the matter and later implemented a variant of the Delphi method in order to reach consensus of an international panel of 51 experts on clinical comunication. A Steering Group (CGL, RRM and FCM) located at the Universidad Francisco de Vitoria (Madrid) took charge of the project organisation and coordination on behalf of the Scientific Committee. The surveys were carried out electronically through a specific website accessible to panelists.

### Preliminary phase of the project: forming the scientific committee and preparing the survey contents

A Scientific Committee (SC) composed of 15 members from Argentina, Brazil, Chile, Colombia, Spain, Mexico and Portugal was created choosing professionals with a leading profile (clinical, teaching or research) on clinical communication in their respectives countries (May 2013). Members included doctors, clinical faculty and other health and non-health related professionals, widely recognized as experts by their professional profile or qualifications (for instance, active membership of subgroup t-EACH (teaching) of the European Association of Communication and Health).

SC members collected and shared any literature on conceptual frameworks, curriculum proposals, teaching guides, training programmes and institutional educational reports from their respective countries, relevant to physician-patient communication teaching and LO for medical studies. A significant number of documents were obtained not only from SC member countries, but also from other Latin American countries not represented in SC [30–55]. A selection of the information was disseminated to all SC members together with a key set of international articles and consensus documents [8–15, 37]. The SC members were asked to review and analyse this material and to make comments and their own proposal of communication LO. With all the information received, a first expanded list of learning outcomes LO was drawn up.

**Fig. 1:**
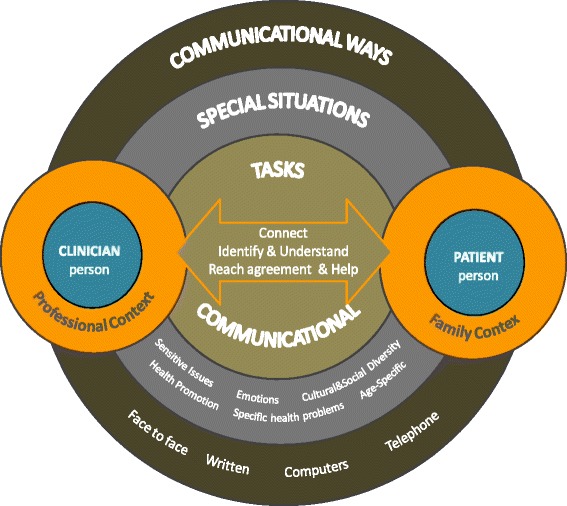
Relevant dimensions in doctor-patient communication

At this stage, the SC members also discussed a conceptual proposal on person-centred care that was realistic for clinical practice and applicable for teaching in our context [55]. This proposal identifies and highlights several dimensions or competency domains considered relevant in the context of healthcare relationships (Fig. 1). The final version obtained from this discussion was used for clustering and hierarchizing the LO subject to consensus [54]. The LO were phrased according to Bloom taxonomies [56] and defined as skills that are demonstrable, focusing on observable results [57]. SC members also received information on what was considered a LO, specifying how they should be worded.

Thus, a first draft of 157 LO was obtained and grouped under 6 general competency domains. SC members discussed this first version and proposed possible changes. The set of proposals for modification, deletion or addition of new LO or relocation of LO within the communication domains as well their supporting comments (227) were received by the Steering Group. A synthesis of these inputs was returned up to three times to the SC members, who discussed and approved a final version with 143 items, which was used to start the Delphi survey.

### Selection of the international expert panel (October 2013)

Eligible experts, i.e. individuals who are widely recognised by peers for their extensive knowledge, (“epistemic” expertise), and/or performance (“performative” expertise) [58] were sought. For the selection of experts, a *snowball sample technique* was used [59] starting from the personal contacts of the SC members. The new “eligible experts” identified were also asked to nominate additional candidates. Thus, a list of 104 “eligible experts” were drawn up. In order to reduce the final size of the panel and select the most suitable candidates, each “eligible expert” was asked to choose from this list, those they considered as most renowned in this field (in relation to specific aspects such as academic leadership, impact publications, research experience, etc.) and to classify each “eligible expert” into one of the following three categories: unquestionable expert, possible expert and without opinion on the candidate’s expertise. Focusing especially on those that received classification as unquestionable or possible expert from several different institutions/countries, the final panel was constituted, including 51 experts from Argentina, Brazil, Chile, Colombia, Spain, Mexico, Peru and Portugal: 37 physicians (4 planners and/or healthcare managers in public and private medical institutions and 1 representative of an organisation for patients’ defence), 5 psychologists and 2 pharmacists, 1 philosopher and 1 PhD in Social Sciences.

### Delphi survey phase

The “modified” Delphi technique [60] avoids the iteration of an excessive (and undetermined) number of survey rounds to achieve stability in groupal responses, allowing a free exchange of views in a face-to-face meeting celebrated between two sole confidential surveys. Although this method is more feasible than the original Delphi method [61], the local meeting defeats one of its cornerstones (anonymity of participants to prevent bias). To avoid this, we used a widely spread and useful variant of the modified Delphi technique with the following characteristics: limited number of rounds (two), no face-to-face encounter, participation of the panel members facilitated by providing detailed feedback between rounds and anonymous survey scores and written views of each participant [62–64]. Between the two rounds, panelists were informed about the group’s responses to the first survey (statistical and graphical description) and received anonymous comments and clarifications that each participant had made. After reviewing this information, they participated in the second round in which they made further assessment on those LO that did not reach consensus in the first round. The electronic circulation of surveys accelerated the process and facilitated monitoring of those who did not reply on schedule. Close follow-up by the technical office was paramount in the process. This process took 12 weeks (October to December 2013). (Additional file 1 with COREQ checklist, providing more methodological details).

### Analysis of the consensus degree

#### Quantitative analysis

The panelists assessed each LO using an ordinal 9 level Likert scale, according to the format developed at UCLA-Rand Corporation [65]. The response categories on this scale are described by semantic descriptors in three regions (1–3 = “disagree”; 4–6 = “neither agree nor disagree”; 7–9 = “agree”). For each LO, the respondent could define their individual opinion in detail, by choosing one of the nine available points. To analyse the group opinion and type of consensus reached, we used the position of the median scores (in which region) and the level of agreement reached by the respondents. Thus, the median score determined the group opinion as “disagreement” on the LO if the median ≤ 3, “agreement” if the median ≥ 7. If the median score was in the 4–6 region, the group opinion was considered “doubtful”. Consensus about one LO was defined when there was panel concordance: that is, when the number of experts scoring outside the region containing the median (the outliers), was less than a third of the respondents. Panel discordance was defined when the scores of a third or more of the panelists were in the region [1–3], and another third or more in the region [7–9]. In those LO where there was neither panel concordance nor discordance, the group opinion was considered as “undetermined”. Any LO which did not obtain either a favourable or unfavourable consensus (ie all LO with doubtful, discordant and undertermined opinions), was included in the second Delphi round for re-assessment by the panel. Any LO that no obtained consensus, or obtained a favourable consensus but more than 25 % respondents’ scores outside the region including median was defined as inappropriate.

#### Qualitative analysis

All comments made by panelists during the Delphi survey were analysed and a list of codes (or categories) was derived inductively from the comments. Three reviewers (RRM, CGL and FCM) independently analysed the comments in more detail and encoding all comments, grouping them in these initial categories or creating new ones. Discrepancies arising from this coding were resolved by verbal agreement between reviewers.

## Results

46 (90 %) experts concluded the two rounds of the Delphi survey. During this process, the panelists made 734 comments (695 for the first round and 39 for the second). At the end of the process, consensus was obtained for 136 (95.1 %) LO. Table 1 (original Spanish and Portuguese version in Additional files 2 and 3) displays these LO, detailing for each one, the mean score, the median, the number of outliers (expressed as a percentage of all respondents) and the interquartile range distribution of scores. Table 2 describes the 34 LO with a mean score greater than 8.34 (the highest 25 %). Most of these items had a maximum of 5 % of experts who disagreed; items 11, 103, 104, 105 and 128 had almost 7 % who disagreed, and in items 12, 30, 43, 62, 63, 74 and 87, 100 % of panelists agreed. The LO which were defined as inappropriate (no obtained consensus, or obtained a favourable consensus but more than 25 % respondents’ scores outside the region including median) are shown in Tables 3 and 4 shows the evolution of objectives from the initial proposal during the Delphi process. Except for category E (Communication through different channels) where a higher percentage of outcomes (57 %) had a level of discordance of between 10 and 20 %, all domains of competence got a high concordance among experts.

**Table 1:** Expert consensus of content (*learning outcomes*) for a communication skills core curriculum for undergraduate medical students

A) Communication with patients (diada)	$\overline{\mathrm{x}}$	M	IQR	% out M
A.1. General Aspects of Clinical Interviews with patients (The student recognises the value of medical interviews for clinical purposes; knows, integrates and structures the various components)				
The student…				
1. Explains the principles and characteristics of human communication.	7,4	7,5	2,0	13,6
2. Explains the professional and patient relationship models (focused on the professional, on the patient, on tasks, on the process, mixed…).	8,0	8,0	2,0	2,3
3. Describes the various content elements of a *patient history* (medical history: *illness* and *disease*, physical and complementary exams, diagnostic approach, care plan, evolution).	8,1	9,0	1,0	6,8
4. Describes the various useful procedural elements for preparing a *patient history* (communication or relational skills).	7,7	8,0	2,0	13,6
5. Delimits the structure of a *clinical interview* from the beginning to the end (introduction, initiating the interview, sharing information: gathering and giving it, planning, setting up next meeting, closing the interview).	8,1	9,0	1,0	9,1
6. Identifies the aspects of doctor-patient communication that have been proven efficient in scientific studies (due to the positive relationship with the outcome of the care).	8,0	8,0	2,0	9,1
7. Recognises the mechanisms through which clinical communication generally leads to improved care outcomes through intermediate outcomes.	7,5	8,0	1,0	13,6
8. Conducts a clinical interview by integrating the content (medical history, exam, diagnosis, care plan and evolution) with the process (communication or relational skills).	8,7	9,0	0,0	2,3
9. Demonstrates acceptance of the importance of the relational context in which the clinical interview is conducted by using adequate behaviours.	8,1	9,0	1,5	9,1
10. Demonstrates a willingness to involve the patient in the interaction, establishing a therapeutic relationship using a patient-centred approach.	8,6	9,0	1,0	2,3
A.2. Tasks and Skills for communication with patients				
A.2.1. Establishes and maintains a therapeutic relationship (Connects) (The student establishes and maintains a therapeutic relationship through a patient-centred approach)				
The student…				
11. Knows the most relevant aspects of non-verbal communication (eye contact, gestures, facial expressions, proxemics, paralanguage…) and their influence on the establishment of an effective relationship.	8,4	9,0	1,0	6,8
12. Verifies the patient feels attended and listened to using techniques such as active listening, questions, checks, etc.	8,8	9,0	0,0	0,0
13. Perceives the patient’s non-verbal language and responds adequately given the context.	8,6	9,0	1,0	2,3
14. Uses clinical history records (manual/computerised) when communicating with the patient in a way that does not interfere.	8,1	9,0	1,0	6,8
15. Applies social communication skills to welcome patients that foster an effective relationship (greeting, calling the patient by name, making them feel comfortable…).	8,7	9,0	0,0	2,3
16. Applies social communication skills to take leave of patients that foster an effective relationship (saying goodbye, accompanying them to the door…).	8,7	9,0	0,0	2,3
17. Demonstrates empathy at the right times (emotional reactions, difficult situations…).	8,6	9,0	0,5	2,3
18. Recognises difficult situations and communication challenges (crying, strong emotions, interruptions, aggression, anger, anxiety, sensitive or embarrassing topics, cognitive difficulties, bad news, first meeting…).	8,6	9,0	1,0	4,6
19. Uses techniques to sensitively and constructively handle difficult situations and communication challenges.	8,4	9,0	1,0	4,6
20. Relates with the patient in a respectful manner considering their rights (confidentiality, privacy, autonomy, respect for their values and beliefs).	8,8	9,0	0,0	2,3
21. Considers the patient as a collaborator in building the relationship and treats the patient as such.	7,8	9,0	1,5	13,6
22. Demonstrates genuine interest in the relationship with the patient and their situation.	8,1	9,0	1,5	9,1
23. Adequately uses a sense of humour in the relationship with the patient (in situations that need calming, to show proximity…).	7,2	8,0	3,0	27,3
A.2.2. Exchange Information and Understand it				
A.2.2.1. Gather information (The student gathers the relevant information to make reasoned clinical decisions)				
The student…				
24. Differentiates *illness* from *disease*, recognising the importance of exploring both perspectives.	8,4	9,0	1,0	2,3
25. Recognises the advantages and disadvantages of various communication skills when gathering information (open/closed questions, eliciting…).	8,2	9,0	1,5	6,8
26. Accurately establishes the reasons for the patient’s visit (open question, no interruptions, explores different reasons…).	8,4	9,0	1,0	4,6
27. Examines and gathers the content of the patient’s bio-psycho-social history (somatic, mental, psychological, family, work…) when the situation requires.	8,3	9,0	1,0	6,8
28. Adds any other element of interest from a people-centred medical approach to the clinical history (spiritual needs, financial difficulties, interferences with leisure time…) which are not commonly recorded on history forms.	8,2	8,5	1,0	6,8
29. Uses different types of questions (open, closed and guided…) as appropriate for each situation.	8,1	9,0	1,0	9,1
30. Uses verbal and non-verbal active listening techniques (reflection, picks up on clues from the patient, paraphrasing, eliciting, summarising…).	8,6	9,0	1,0	0,0
31. Briefly repeats the information gathered to the patient for verification.	8,1	9,0	2,0	13,6
32. Assesses how the illness affects the patient’s daily life, socio-family environment and work environment.	8,5	9,0	1,0	4,6
33. Considers other factors that can influence a patient’s needs when enquiring (ideas, fears, feelings, preferences, prior experiences…).	8,5	9,0	1,0	2,3
34. Establishes adequate support for the physical exam (requesting permission, explaining what is going to be done and why, sharing findings with the patient…).	8,5	9,0	1,0	2,3
35. Recognises the divergences between medical and a patient’s values and standards, respecting them without judgement.	8,4	9,0	1,0	2,3
36. Demonstrates openness and a willingness to appropriately handle any aspect that is important to the patient as concerns healthcare using appropriate behaviours.	7,9	8,0	2,0	6,8
A.2.2.2. Give information (The student gives the information the patient needs to make decisions in a way that is clear and personalised)				
The student…				
37. Critically assess the scientific findings on transferring information to patients and the implications in clinical practice.	7,9	8,0	2,0	11,4
38. Describes the basic principles of adequately informing patients of risks (avoiding any type of manipulation and/or partiality when presenting figures and likelihoods…).	8,3	9,0	1,0	6,8
39. Communicates the risk to the patient, making personalised use of the indicators (risk measures).	7,7	8,0	2,0	18,2
40. Supplements this verbal information with diagrams, models, written information and instructions, when necessary.	8,1	8,5	1,0	11,4
41. Estimates the patient’s level of knowledge of their problem and to what extent the patient wishes to be informed to provide the information the patient actually requires.	8,5	9,0	1,0	2,3
42. Gives the patient the information properly (adequate circumstance).	8,3	9,0	1,0	6,8
43. Adapts communication to the patient’s level of understanding and language, avoiding any medical jargon.	8,7	9,0	0,0	0,0
44. Provides patient-centred information from their perspective and making it meaningful for the patient.	8,4	9,0	1,0	4,6
45. Discusses benefits, risks and expected outcomes in a patient-centred manner.	8,1	9,0	1,0	9,1
46. Verifies the patient understands the information provided by eliciting any questions.	8,6	9,0	0,0	2,3
47. Explains precise information to the patient to minimise any uncertainty when making decisions.	8,1	9,0	1,0	6,8
48. Shares the information with third parties (colleagues, family and others) with the patient’s consent.	8,0	9,0	2,0	15,9
A.2.3. Make joint decisions and Help the patient carry out what was decided (The student makes decisions considering the patient’s participation and responsibility as well as their preferences)				
The student…				
49. Differentiates the various ways patients can participate in the decision making (paternalism, consumerism, collaborative…).	7,3	8,0	2,5	25,0
50. Establishes what should be the most appropriate physician role in the decision making process with each patient.	7,8	8,0	2,0	13,6
51. Accepts the role of uncertainty as a substantial element in clinical reasoning and decision making.	7,9	9,0	2,0	13,6
52. Recognises the elements that contribute to the presence of uncertainty (lack of professional knowledge, lack of evidence…) in clinical decision making.	7,6	8,5	2,0	20,5
53. Communicates the fact that there is uncertainty to the patient in a way that adapts to the patient’s level of tolerance.	7,8	8,0	2,0	22,7
54. Explores the patient’s needs, resources (information, autonomy, trust, responsibility, psychological features…) and willingness to get them involved in the decision making.	8,3	9,0	1,0	4,6
55. Reaches agreements with the patient using negotiation skills.	8,0	9,0	1,5	6,8
56. Understands the role of aids in clinical practice when making decisions (decision aids).	6,9	7,5	2,0	29,6
57. Teaches the patient about using decision aids so they may be used in the discussion.	6,9	8,0	3,0	29,6
58. Clarifies how and when the decision must be made with the patient.	7,7	8,0	2,0	15,9
59. Discusses the spectrum of possible consequences of a decision with the patient (explains the consequences of choosing/not choosing the option discussed).	8,0	8,0	1,0	6,8
60. Offers the patient the option of opening up and enriching the decision-making discussion by including third parties (colleagues, family members).	7,9	8,0	2,0	11,4
61. Uses informed consent in a way that the patient understands the characteristics and consequences of the procedure.	8,3	9,0	1,0	6,8
62. Adapts the plan/treatment to the patient’s resources and strengths.	8,5	9,0	1,0	0,0
63. Closes the process at the end of the visit by using the adequate communication strategies (summarising, highlighting key aspects, anticipating possible evolutions and offering guidance…).	8,7	9,0	0,5	0,0
64. Assumes the patient’s involvement and responsibility in the decision-making process and uses adequate behaviours in doing so.	7,8	8,0	2,0	20,5
65. Is willing to re-assess and review own decisions.	8,2	9,0	1,0	9,1
B) Communication with the patient’s family	$\overline{\mathrm{x}}$	M	IQR	% out M
B.1. The patient’s family context (The student recognises and assesses the family’s role in the patient’s clinical care and establishes effective communication with the family in patient’s benefit)				
The student…				
66. Knows the family’s role as a system in patient care.	8,2	9,0	1,5	9,1
67. Describes the basic models that explain family and patient behaviour as if one of the members (the individual and family life cycle).	7,6	8,0	2,0	20,5
68. Knows and uses methods and tools to identify the family structure and functioning (genograms or family trees, family function, stressful events, social network…).	7,2	7,0	3,0	27,3
69. Considers the family’s response pattern to the disease and the stressful life events when caring for a patient.	7,6	8,0	2,0	15,9
70. Identifies the family member(s) who fulfil the role of main caretaker in order to involve them in the process and assess them in this role.	7,9	9,0	1,0	11,4
71. Requests and synthesizes relevant information from other family members and patient caretakers, if necessary and they are available.	8,0	8,0	1,5	9,1
72. Establishes effective communication with the patient and their family to identify problems, detect resources and implement action plans that benefit the patient.	8,4	9,0	1,0	2,3
73. Helps the family make decisions when the patient is a minor or incapacitated (dementias, patients in a coma, incapacitating mental illnesses…).	8,5	9,0	1,0	2,3
74. Recognises specific family-related communication challenges (confidentiality, secrecy, ill companions…).	8,4	9,0	1,0	0,0
75. Demonstrates a willingness to include the family and work with them for the patient’s benefit.	8,3	9,0	1,5	4,6
76. Demonstrates a willingness to ease communication among the patient’s family members by using adequate behaviours.	7,6	8,5	2,0	13,6
77. Demonstrates sensitivity toward the family’s fears and concerns, using adequate behaviours.	8,3	9,0	1,0	2,3
C) Intra-personal communication (self-perception)	$\overline{\mathrm{x}}$	M	IQR	% out M
C.1. Doctors as people (self-awareness, self-reflection, self-criticism, self-care) (The student habitually reflects upon their own behaviour and means of communication, developing and improving their self-awareness, self-reflection, self-criticism, self-care)				
The student…				
78. Describes the factors that influence the relationship between the professional and the patient (stereotypes, sociocultural prejudices, experiences, interests…).	7,9	8,5	2,0	15,9
79. Critically reflects upon their own communication and behavioural style, considering possible alternatives.	8,3	9,0	1,0	4,6
80. Recognises the barriers that make self-awareness difficult and uses techniques and strategies to foster it such as reflection, a personal perspective…	8,3	9,0	1,0	6,8
81. Identifies the signs of work and stress overload (insomnia, anxiety, sleep alterations…).	8,3	9,0	1,0	4,6
82. Distinguishes the main sources of medical errors (deficient information or assessment of patient’s needs, inadequate communication…).	8,1	9,0	1,5	4,6
83. Recognises the technical errors, cognitive bias and emotional reactions that hinder the development of therapeutic relationships.	8,2	9,0	1,0	4,6
84. Uses strategies to reduce stress and overload (relaxation, focus groups, Balint groups, supervision and support…).	7,8	9,0	2,0	18,2
85. Controls own emotional reactions and works efficiently even in difficult situations (high degree of patient suffering, a demanding patient…).	8,0	9,0	1,0	11,4
86. Develops the necessary mental habits to recognise own biases through the use of specific techniques (thought-provoking questions, perspective observation, *mindfulness*, suspension of judgement, non-prejudiced attitudes…).	8,0	8,0	2,0	6,8
87. Recognises own errors (and those of others), assumes them as a part of the job and seeks solutions for them (assistance from superiors…).	8,5	9,0	1,0	0,0
88. Recognises own emotions (insecurity, sympathy/antipathy, attraction…) in relation to others (patients, colleagues).	8,1	9,0	1,0	6,8
89. Values certain self-perceived personal strengths and weaknesses in the proper teaching contexts (guidance, mentorship, focus groups…).	8,2	8,5	1,0	6,8
90. Accepts and approaches own uncertainty in a way that is adequate to the educational stage.	7,7	8,0	2,0	13,6
D) Inter-intra professional communication	$\overline{\mathrm{x}}$	M	IQR	% out M
D.1. The professional medical context: Inter- and intra-personal communication (The student communicates efficiently with the professionals within or outside their team)				
The student…				
91. Identifies the basic principles of group dynamics as well as supporting and inhibiting factors.	7,7	8,0	2,0	15,9
92. Identifies the different members of the various inter-professional healthcare teams and their respective responsibilities.	8,0	9,0	1,0	9,1
93. Clarifies own role and responsibilities as a student in the professional teams with which they interact.	8,1	9,0	1,0	11,4
94. Identifies when to seek assistance from professionals/institutions/agencies that can help solve a given problem.	8,2	9,0	1,5	6,8
95. Describes the principles and strategies for negotiating and resolving conflicts with other professionals and uses them adequately.	7,5	8,0	2,0	22,7
96. Discusses decisions appropriately with colleagues, patients and family members and, if necessary, re-assesses own decisions.	8,2	9,0	1,0	9,1
97. Ensures all of the patient’s relevant clinical information is available.	7,4	8,5	2,0	18,2
98. Facilitates the flow of information concerning opinions in the group and encourages the members of the team to offer divergent opinions.	7,9	9,0	2,0	13,6
99. Gives feedback to members of the team in an appropriate manner (first-person comments, highlights the positive aspects first, does not judge).	8,0	9,0	2,0	9,1
100. Effectively contributes to care continuity when patients are referred and returned to and from different care levels (primary, specialised).	7,8	8,5	2,0	13,6
101. Makes clinical or scientific presentations in public effectively.	7,6	8,0	2,0	15,9
102. Gives instructions clearly and precisely.	8,3	9,0	1,0	9,1
103. Helps create a positive working atmosphere (supports and includes the different members of the team, mentions the positive side of unpleasant aspects, values the team’s success…).	8,4	9,0	1,0	6,8
104. Respects individuality, the subjective perspectives of the members of the team and the mastery (expert skills) of the various healthcare professionals.	8,4	9,0	1,0	6,8
105. Maintains confidentiality when making decisions as a team.	8,5	9,0	1,0	6,8
106. Is assertive with the rest of the members of the team.	7,8	8,0	2,0	13,6
107. Demonstrates a negotiating attitude in order to reach agreements, using adequate behaviours.	8,2	8,0	2,0	4,6
108. Demonstrates flexibility in changing roles within a team.	8,3	9,0	1,0	4,6
E) Communication through different channels				
E.1. Communication channels (The student efficiently uses the various means of communication)				
E.1.1. Direct communication (face-to-face)	$\overline{\mathrm{x}}$	M	IQR	% out M
The student…				
109. Identifies environmental factors (physical and social) that can hinder interpersonal communication in different contexts.	8,3	9,0	1,0	4,5
110. Identifies whether there is any discrepancy between the verbal and non-verbal components of communication.	8,2	9,0	1,5	9,1
111. Adequately uses proxemics (physical distance when communicating).	8,2	9,0	1,0	6,8
E.1.2. Written communication				
The student…				
112. Recognises the clinical history forms and resources and the documents habitually used for written communication with patients and among professionals (discharge reports, referrals, test requests…).	8,1	9,0	1,5	13,6
113. Records the initial assessment of a patient as well as any later daily clinical evolution in concise and clear written language.	8,1	9,0	1,0	13,6
114. Writes a structured, comprehensible, sufficient and clear discharge report and referral.	8,2	9,0	1,0	11,4
115. Writes tests requests and prescriptions precisely, clearly and in a justified manner.	8,0	9,0	2,0	11,4
116. Maintains clear and appropriate records on relevant information relating to the clinical meeting.	8,2	9,0	1,0	9,1
117. Writes the habitual legal documents (death certificates, health certificates…).	8,3	9,0	1,0	6,8
E.1.3. Electronic or computerised communication				
The student…				
118. Recognises the various information technologies most often used in healthcare.	7,7	8,0	2,0	15,9
119. Is aware of the patients’ electronic records as well as the prescription and referral systems.	8,0	8,0	2,0	6,8
120. Handles information technologies (emails, WhatsApp, web 2.0…) for healthcare issues in a way that ensures confidentiality.	7,9	8,0	2,0	15,9
E.1.4. Telephone communication				
The student…				
121. Recognises the uses and limits of telephone communication with patients.	8,0	9,0	2,0	11,4
122. Communicates with patients by telephone, attending to their specific requests and the communication adaptations this channel requires.	7,8	8,0	2,0	13,6
F) Communication in special situations				
F.1. Specific communication contests (The student applies and adapts the core communication skills in specific clinical situations and uses the specific skills required in each situation)				
F.1.1. Sensitive situations	$\overline{\mathrm{x}}$	M	IQR	% out M
The student…				
123. Recognises delicate situations that represent communication challenges (giving bad news, handling end-of-life topics, grief situations, sexual history, gender violence, child abuse, HIV infection, explaining situations of clinical uncertainty…).	8,5	9,0	1,0	4,5
124. Approaches some of them in a sensitive and constructive manner by applying and adapting the core communication skills and using the specific strategies and skills that each may require.	8,2	9,0	1,0	9,1
125. Is aware of the essential legal aspects in effect in each jurisdiction relating to how some of these situations must be handled.	7,9	9,0	2,0	13,6
F.1.2. Managing emotions				
The student…				
126. Recognises situations of emotional tension during visits (stress, fear, anger, aggressiveness, denial, collusion, embarrassment…).	8,3	9,0	1,0	4,5
127. Approaches some of them in a sensitive and constructive manner by applying and adapting the core communication skills and using the specific strategies and skills that each may require.	7,9	8,5	1,5	9,1
F.1.3. Cultural and social diversity				
The student…				
128. Recognises patients’ cultural and social diversity (ethnicity, nationality, socio-economic status, language, religion, gender, values, sexuality…) and the communication difficulties involved.	8,4	9,0	1,0	6,8
129. Approaches some of them in a sensitive and constructive manner by applying and adapting the core communication skills and using the specific strategies and skills that each may require.	8,0	8,5	1,0	13,6
F.1.4. Health promotion and behaviour change				
The student…				
130. Describes the basic principles of motivation.	7,7	8,5	2,0	13,6
131. Recognises the stages of change a patient is in when modifying behaviours or following treatments.	8,0	8,5	2,0	9,1
132. Explores the patient’s level of motivation for change.	8,2	9,0	1,5	6,8
133. Applies effective communication strategies to change behaviours.	8,1	9,0	1,0	11,4
134. Assumes a prevention and health promotion approach when caring for patients and uses adequate behaviours.	8,4	9,0	1,0	2,3
F.1.5. Specific clinical contexts				
The student…				
135. Approaches some specific clinical contexts (psychiatric, patients with dementia, with sensory problems: hearing, visual, verbal expression) by applying and adapting the core communication skills and using the specific strategies and skills each of them require.	8,1	9,0	2,0	11,4
F.1.6. Patients of different ages				
The student…				
136. Communicates with patients of different age groups (children and parents, adolescents, the elderly) by applying and adapting the core communication skills and using the specific strategies and skills that each one may require.	8,3	9,0	1,5	2,3

$\overline{\mathrm{x}}$ Mean, *M* Median, *IQR* Interquartile range, *% out the M* Percentage of responders outside the region including median

**Table 2:** Items agreed upon with highest mean score (25 % superior, mean score > 8.34)

N° item	CAT		$\overline{\mathrm{x}}$	M	IQR	% out M
8	A	Conducts a clinical interview by integrating the content (medical history, exam, diagnosis, care plan and evolution) with the process (communication or relational skills).	8,7	9,0	0,0	2,3
10	A	Demonstrates a willingness to involve the patient in the interaction, establishing a therapeutic relationship using a patient-centred approach.	8,6	9,0	1,0	2,3
11	A	Knows the most relevant aspects of non-verbal communication (eye contact, gestures, facial expressions, proxemics, paralanguage…) and their influence on the establishment of an effective relationship.	8,4	9,0	1,0	6,8
12	A	Verifies the patient feels attended and listened to using techniques such as active listening, questions, checks, etc.	8,8	9,0	0,0	0,0
13	A	Perceives the patient’s non-verbal language and responds adequately given the context.	8,6	9,0	1,0	2,3
15	A	Applies social communication skills to welcome patients that foster an effective relationship (greeting, calling the patient by name, making them feel comfortable…).	8,7	9,0	0,0	2,3
16	A	Applies social communication skills to take leave of patients that foster an effective relationship (saying goodbye, accompanying them to the door…).	8,7	9,0	0,0	2,3
17	A	Demonstrates empathy at the right times (emotional reactions, difficult situations…).	8,6	9,0	0,5	2,3
18	A	Recognises difficult situations and communication challenges (crying, strong emotions, interruptions, aggression, anger, anxiety, sensitive or embarrassing topics, cognitive difficulties, bad news, first meeting…).	8,6	9,0	1,0	4,6
19	A	Uses techniques to sensitively and constructively handle difficult situations and communication challenges.	8,4	9,0	1,0	4,6
20	A	Relates with the patient in a respectful manner considering their rights (confidentiality, privacy, autonomy, respect for their values and beliefs).	8,8	9,0	0,0	2,3
24	A	Differentiates illness from disease, recognising the importance of exploring both perspectives.	8,4	9,0	1,0	2,3
26	A	Accurately establishes the reasons for the patient’s visit (open question, no interruptions, explores different reasons…).	8,4	9,0	1,0	4,6
30	A	Uses verbal and non-verbal active listening techniques (reflection, picks up on clues from the patient, paraphrasing, eliciting, summarising…).	8,6	9,0	1,0	0,0
32	A	Assesses how the illness affects the patient’s daily life, socio-family environment and work environment.	8,5	9,0	1,0	4,6
33	A	Considers other factors that can influence a patient’s needs when enquiring (ideas, fears, feelings, preferences, prior experiences…).	8,5	9,0	1,0	2,3
34	A	Establishes adequate support for the physical exam (requesting permission, explaining what is going to be done and why, sharing findings with the patient…).	8,5	9,0	1,0	2,3
35	A	Recognises the divergences between medical and a patient’s values and standards, respecting them without judgement.	8,4	9,0	1,0	2,3
41	A	Estimates the patient’s level of knowledge of their problem and to what extent the patient wishes to be informed to provide the information the patient actually requires.	8,5	9,0	1,0	2,3
43	A	Adapts communication to the patient’s level of understanding and language, avoiding any medical jargon.	8,7	9,0	0,0	0,0
44	A	Provides patient-centred information from their perspective and making it meaningful for the patient.	8,4	9,0	1,0	4,6
46	A	Verifies the patient understands the information provided by eliciting any questions.	8,6	9,0	0,0	2,3
62	A	Adapts the plan/treatment to the patient’s resources and strengths.	8,5	9,0	1,0	0,0
63	A	Closes the process at the end of the visit by using the adequate communication strategies (summarising, highlighting key aspects, anticipating possible evolutions and offering guidance…).	8,7	9,0	0,5	0,0
72	B	Establishes effective communication with the patient and their family to identify problems, detect resources and implement action plans that benefit the patient.	8,4	9,0	1,0	2,3
73	B	Helps the family make decisions when the patient is a minor or incapacitated (dementias, patients in a coma, incapacitating mental illnesses…).	8,5	9,0	1,0	2,3
74	B	Recognises specific family-related communication challenges (confidentiality, secrecy, ill companions…).	8,4	9,0	1,0	0,0
87	C	Recognises own errors (and those of others), assumes them as a part of the job and seeks solutions for them (assistance from superiors…).	8,5	9,0	1,0	0,0
103	D	Helps create a positive working atmosphere (supports and includes the different members of the team, mentions the positive side of unpleasant aspects, values the team’s success…).	8,4	9,0	1,0	6,8
104	D	Respects individuality, the subjective perspectives of the members of the team and the mastery (expert skills) of the various healthcare professionals.	8,4	9,0	1,0	6,8
105	D	Maintains confidentiality when making decisions as a team.	8,5	9,0	1,0	6,8
123	F	Recognises delicate situations that represent communication challenges (giving bad news, handling end-of-life topics, grief situations, sexual history, gender violence, child abuse, HIV infection, explaining situations of clinical uncertainty…).	8,5	9,0	1,0	4,5
128	F	Recognises patients’ cultural and social diversity (ethnicity, nationality, socio-economic status, language, religion, gender, values, sexuality…) and the communication difficulties involved.	8,4	9,0	1,0	6,8
134	F	Assumes a prevention and health promotion approach when caring for patients and uses adequate behaviours.	8,4	9,0	1,0	2,3

$\overline{\mathrm{x}}$ Mean, *M* Median, *IQR* Interquartile range, *% out the M* Percentage of responders outside the region including median

**Table 3:** Items not agreed/agreed with more than 25 % of experts on against

N° item	CAT		$\overline{\mathrm{x}}$	M	IQR	% out M
NC1	C	Use the systemic model to approach families	6,1	7	6	37,2
NC2	D	Identify and apply basic leadership skills	6,5	7	4	39,5
23	A	Adequately uses a sense of humour in the relationship with the patient (in situations that need calming, to show proximity…).	7,2	8,0	3,0	27,3
56	A	Understands the role of aids in clinical practice when making decisions (decision aids).	6,9	7,5	2,0	29,6
57	A	Teaches the patient about using decision aids so they may be used in the discussion.	6,9	8,0	3,0	29,6
68	B	Knows and uses methods and tools to identify the family structure and functioning (genograms or family trees, family function, stressful events, social network…).	7,2	7	3	27,3

$\overline{\mathrm{x}}$ Mean, *M* Median, *IQR* Interquartile range, *% out the M* Percentage of responders outside the region including median

**Table 4:** Evolution of items from the original proposal during Delphi process for each category

Categoríes	n° ítems initial proposal	n° ítems pre-delphi	1^st^ round not agreed	1^st^ round removed	1^st^ round agreed ítems	2^st^ round agreed items
a) Communication with the patient	78	66		1	65	65
b) Communication with the patient’s family	11	13	1		12	12
c) Communication intrapersonal	18	14		1	13	13
d) Inter-intra professional communication	22	20	1	1	18	18
e) Communication by different routes	15	16		2	14	14
f) Communication in special situation	13	14			14	14
	157	143	2	5	136	136

### Qualitative analysis

All panelists made comments during the Delphi process and by the end of the survey, 734 comments had been received (695 in the 1st round and 39 in the second) and were classified into 14 categories. Most of the comments highlighted the relevance and importance of the LO to be included in undergraduate medical curricula. Other comments were related to: (i) Practical implementation of LO in undergraduate teaching, (ii) Clarification of the LO meaning, avoiding cultural misunderstanding, (iii) Non-exclusive communication nature of LO (iv) LO related to the clinical experience and personal maturity of the apprentice, (v) Implications for teaching and assessment methods, (vi) Appropriateness of learning domain.

## Discussion and conclusion

### Discussion

The LAPS-CCC consensus reached in this study has 136 LO grouped into 6 domains considered important for clinical communication. This consensus was created by 46 experts from 8 Spanish and Portuguese-speaking countries and refers to *“nuclear communication learning outcomes for medical students”*. This consensus is intended as a proposal for the medical schools in LAPS cultural context to be used to facilitate the designing and development of their own clinical communication curricula. The review and discussion of literature, especially that provided by the SC members relative to their countries, confirmed the importance of creating a LAPS consensus: there is a lack of guidelines for communication teaching in LAPS, many universities do not an explicit curriculum for communication or are just developing communication skills teaching and there is great diversity in how communication is being taught.

As in other consensus proposals that inspired this work [12–15], this consensus proposal considers that effective clinical communication is determined by those communicational skills related directly with the physician-patient interaction, as well as skills to communicate with the family, teamwork, and for reflective practice. Communicating through various communication channels and in specific contexts also requires specific skills [13, 54]. The grouping of the LO into these domains reveals some of the particular features of this proposal. The higher number of LO [65] included in the general “patient communication” competency domain points to the importance of patient-centred care: many of these LO are related to communication tasks that have been shown to be important in helping students understand the purpose of the medical consultation and in achieving consultation goals in clinical practice [9–13]. Another distinguishing aspect of our consensus is the numer of LO [14] related to “communication with the family”. Although there are significant differences in the role of the family in the different LAPS countries depending on the degrees of “Europeanization” or the type of community (indigenous, rural, urban) the values of loyalty, respect, solidarity and reciprocity among family members are deeply rooted in the LAPS cultures and have been contrasted with North European and North American individualism. This implies the need to emphasize specific aspects of physician-patient/family relationship that this consensus reflects to a greater extent than others [66, 67].

Most LO in the proposal are more attitudinal and behavioral than cognitive and to incorporate these LO into the medical curriculum will entail a number of practical challenges for our institutions. Experiential teaching methods will need to be prioritized [6, 68] and well-structured feedback using reliable and validated tools will be required [69–71]. For students to achieve these LO, they will need repeated exposure to a variety of clinical situations in which they can be observed and have time for learner’s self-reflection. Teaching will need to include not only cognitive-behavioral strategies, but also reflective methods (i.e., reflective small groups, or directed reflection) [72], not as an isolated course, but throughout the curriculum, and with teachers who have been adequately trained [73, 74]. This proposal of LO should be able to help many schools to design and modify their programmes in a more efficient way [27]. However, it is important to note, as many panelists did in their comments, that, despite the importance of introducing these LO, it may not be an easy task for young students, some of whom have little practical experience during the whole of their undergraduate programme. Many of these objectives require the student to grasp subtle aspects of the hidden curriculum and develop intuitive thinking [75], which may only be achieved by a slow incubation period into the *milieu* and frequent practice [76, 77].

With such challenges and the high number of objectives, one could question whether this proposal really defines a “minimum standard” for LAPS medical schools. Perhaps one of the most interesting and useful contributions of this proposal is the set of 34 objectives with the highest degree of agreement that we have called the“core of the core”. Most of these LO (24/34; 70.5 %) belong to the generic competency domain of “doctor-patient communication skills” and its content includes general communication objectives to consider in the process of doctor-patient relationship and skills for the development of an interview. This type of LO are also the most valued in other consensuses [12, 14, 15]: the UK proposal, for example, locates these skills in the third ring of the “Communication Curriculum Wheel” emphasizing them as “the backbone of effective clinical communication curriculum” [13].

The challenge of reaching a consensus on LO in a structured way, incorporating the best expert opinion, and avoiding biases is elusive. Previous similar studies have predominantly used variants of the original Delphi and Nominal Group technique [14, 15, 78]. In this study, we apply a variant of the modified Delphi method, which replaces its traditional face-to-face interaction of the experts by sending them a detailed result report of the first survey (including statistical results, a remembrance of personal position and comments from other panelists in relation to each LO [62–64].

The reliability and validity of the results are conditioned by aspects such as the selection and participation of the experts involved (number of participants, professional profile, level of expertise and type of participation in the process), the type of questionnaire proposed, the definition of a sufficient level of agreement and subsequent applicability [79, 80]. This consensus reflects the views of the 46 subjects, but other people might have reached a different consensus. The key issue here is whether the participants are really authentic subject matter experts and with influence in their respective scopes. The technique used in the study for the selection of experts (*snowball sample*), allows the identification of a potential expert in a more comprehensive way (through an active search through potential experts networks and a consensus involving the re-selection/confirmation of a subset of experts by the experts themselves) than when this is done by direct election of an expert [81]. Thus, a biased selection (by awareness and desirability of the initial members of the SC) should have been mitigated [82]. So, in our study of 104 potential experts located in 9 countries, we selected 51 from 8 countries, and who represent a wide variety of medical specialties and teaching responsibilities; other health-related professions were also included. Although a representative of patients’ associations was included, the presence of a larger number of patients or the inclusion of students may have enriched the panel findings.

There does not appear to be a optimal size for a Delphi panel [83]. Although forecast error decreases as the sample size increases, in general a panel of more than 30 members is not usually efficient [84]. The number of participants in our consensus is greater than in other previous professional consensuses on communication [8], but lower than in the two major existing consensuses [14, 15]. However, our panelists’ participation during the process was higher than in these consensuses and can be considered high (90.2 %; 46/51).

One of the main strengths of the Delphi method lies in its iterative nature, which encourages participants to revisit their opinions and reflect on them once they have known others’. We feel that a highlight of our process was detailed anonymous feedback between rounds.

The extensive review of the literature included an exhaustive review of the most relevant recommendations and proposals in LAPS. Preliminary discussion on these and on the communication domains to consider the structure created an important common ground for the preparation of a list of LO. The criteria used to mark the consensus has been statistical and, though accepted, is a discretionary criterion. It is hard to imagine a better way to standardize and ensure the process and its results due to the risks involved in unstructured discussions [85].

The final validity of this consensus will be determined by the number of institutions and programmes that use it for designing or adapting their own communication curricula. In this sense, the experts involved can also play an important role in disseminating the proposal within their own institutions and in their areas of influence.

### Conclusions

This study presents a core clinical communication curriculum with 136 LO for undergraduate medical students proposed by 46 experts from 8 Spanish and Portuguese-speaking countries reached through a structured technical consensus (variation of modified Delphi). Thirty-four of these LO have a high degree of consensus and could be especially useful as a basic framework for the development of undergraduate medical educational programmes in clinical communication.

### Practical implications

The LAPS-CCC is a proposal that can be useful to increase awareness and disseminate patient-physician communication educational programmes in medical schools in the LAPS countries or in other countries with similar characteristics. The LAPS-CCC can guide the design of communication programmes for medical students, especially regarding the choice of content (LO) that each institution may consider as more suitable for their graduates, based on their priorities and circumstances. This proposal can guide the development of teaching strategies and assessment communication skills in a more efficient way. The LAPS-CCC is proposed as a document of reference for the development of similar initiatives in other health profession studies.

## Additional files

Additional file 1: COREQ checklist. (DOCX 20 kb) Additional file 2: LAPS_CCC Spanish version. (DOCX 44 kb) Additional file 3: LAPS_CCC Portuguese version. (DOCX 36 kb)
