# Supplementary material for: A Latin American, Portuguese and Spanish consensus on a core communication curriculum for undergraduate medical education
Source: BMC Med Educ. 2016 Mar 28;16:99. doi: 10.1186/s12909-016-0610-8 (PMC4809037; doi:10.1186/s12909-016-0610-8)
Supplement: Additional file 1: — COREQ checklist. (DOCX 20 kb) [file 12909_2016_610_MOESM1_ESM.docx]

**“Latin American, Portuguese and Spanish consensus on a Core Communication Curriculum**

**for undergraduate medical education”:** Consolidated criteria (COREQ) for reporting the qualitative research content

The main methodological research method of this study has been a consensus method “Modified Delphi technique”. As a secondary aim

we collected comments to the items (LO) made by pannelists and handled by qualitative analysis

| **No** | **Item** | **Guide questions/description** | **Comments & specific page number where this information can be found** |
| --- | --- | --- | --- |
| **DOMAIN 1:**  **RESEARCH TEAM AND REFLEXIVITY** |  |  |  |
| **Personal Characteristics** |  |  |  |
| 1. | Interviewer/facilitator | Which author/s conducted the interview or focus group? | Garcia de Leonardo C |
| 2. | Credentials | What were the researcher's credentials? *E.g. PhD, MD* | MD |
| 3. | Occupation | What was their occupation at the time of the study? | Academic researcher |
| 4. | Gender | Was the researcher male or female? | Female |
| 5. | Experience and training | What experience or training did the researcher have? | Residency training in Family Medicine and two year of methodological research |
| **Relationship with participants** |  |  |  |
| 6. | Relationship established | Was a relationship established prior to study commencement? | None |
| 7. | Participant knowledge of the interviewer | What did the participants know about the researcher? e*.g. personal goals, reasons for doing the research* | Main academic reasons for carrying out this project |
| 8. | Interviewer characteristics | What characteristics were reported about the interviewer/facilitator? e.g. *Bias, assumptions, reasons and interests in the research topic* | Involved in the UFV Medicine curriculum |
| **DOMAIN 2:**  **STUDY DESIGN** |  |  |  |
| **Theoretical framework** |  |  |  |
| 9. | Methodological orientation and Theory | What methodological orientation was stated to underpin the study? *e.g. grounded theory, discourse analysis, ethnography, phenomenology, content analysis* | Consensus methodology (P 7)  Content analysis for pannelists comments (P8) |
| **Participant selection** |  |  |  |
| 10. | Sampling | How were participants selected? *e.g. purposive, convenience, consecutive, snowball* | Snowball (P 6) |
| 11. | Method of approach | How were participants approached? e*.g. face-to-face, telephone, mail, email* | Email/website accesible only to pannelists (P 5) |
| 12. | Sample size | How many participants were in the study? | 51 (P 6) |
| 13. | Non-participation | How many people refused to participate or dropped out? Reasons? | 5 (P 8)  Do not answer the surveys |
| **Setting** |  |  |  |
| 14. | Setting of data collection | Where was the data collected? e*.g. home, clinic, workplace* | website accesible only to pannelists (P 5) |
| 15. | Presence of non-participants | Was anyone else present besides the participants and researchers? | No |
| 16. | Description of sample | What are the important characteristics of the sample? *e.g. demographic data, date* | P 5-6-7 & Appendix 1 (P15) |
| **Data collection** |  |  |  |
| 17. | Interview guide | Were questions, prompts, guides provided by the authors? Was it pilot tested? | P 5-6; Figure 1; |
| 18. | Repeat interviews | Were repeat interviews carried out? If yes, how many? | Delphi survey of two rounds (P 7) |
| 19. | Audio/visual recording | Did the research use audio or visual recording to collect the data? | No |
| 20. | Field notes | Were field notes made during and/or after the interview or focus group? | No |
| 21. | Duration | What was the duration of the interviews or focus group? | 12 weeks (P 7) |
| 22. | Data saturation | Was data saturation discussed? | NA |
| 23. | Transcripts returned | Were transcripts returned to participants for comment and/or correction? | Yes (P 7) |
| **DOMAIN 3: ANALYSIS AND FINDING** |  |  |  |
| **Data analysis*** |  |  | *Only applicable to the pannelists comments |
| 24. | Number of data coders | How many data coders coded the data? | Three (P 8) |
| 25. | Description of the coding tree | Did authors provide a description of the coding tree? | No |
| 26. | Derivation of themes | Were themes identified in advance or derived from the data? | Derived from the data (P 8) |
| 27. | Software | What software, if applicable, was used to manage the data? | None |
| 28. | Participant checking | Did participants provide feedback on the findings? | Yes (P 7) |
| **Reporting** |  |  |  |
| 29. | Quotations presented | Were participant quotations presented to illustrate the themes / findings? Was each quotation identified? e*.g. participant number* | No (as these are not the primary goal of the paper) |
| 30. | Data and findings consistent | Was there consistency between the data presented and the findings? | NA |
| 31. | Clarity of major themes | Were major themes clearly presented in the findings? | NA |
| 32. | Clarity of minor themes | Is there a description of diverse cases or discussion of minor themes? | Yes (P11) |
