# Supplementary material for: A Latin American, Portuguese and Spanish consensus on a core communication curriculum for undergraduate medical education
Source: BMC Med Educ. 2016 Mar 28;16:99. doi: 10.1186/s12909-016-0610-8 (PMC4809037; doi:10.1186/s12909-016-0610-8)
Supplement: Additional file 2: — LAPS_CCC Spanish version. (DOCX 44 kb) [file 12909_2016_610_MOESM2_ESM.docx]

| **A) COMUNICACION CON EL PACIENTE (DIADA)** **A.1. Aspectos generales de la Entrevista Clínica con pacientes**  (El estudiante reconoce el valor de la entrevista médica para la finalidad del acto clínico conociendo, integrando y estructurando sus diferentes componentes)  **El estudiante será capaz de…** | **X*** | **Me*** | **RI*** | **% fuera Me*** |
| --- | --- | --- | --- | --- |
| 1. Explicar los principios y características de la comunicación humana. | 7,4 | 7,5 | 2,0 | 13,6 |
| 2. Explicar los modelos de relación entre profesional y paciente (centrados en el profesional, en el paciente, en tareas, en el proceso, mixtos…). | 8,0 | 8,0 | 2,0 | 2,3 |
| 3. Describir los diferentes elementos de contenido de una historia clínica (anamnesis: dolencia (*illness*) y enfermedad (*disease*), exploración física y complementaria, aproximación diagnóstica, plan terapéutico, evolución). | 8,1 | 9,0 | 1,0 | 6,8 |
| 4. Describir los diferentes elementos de proceso útiles para la elaboración de una historia clínica (habilidades comunicacionales o de relación). | 7,7 | 8,0 | 2,0 | 13,6 |
| 5. Delimitar la estructura de una entrevista clínica desde su inicio hasta su final (introducción, iniciar la entrevista, compartir información: obtenerla y ofrecerla, planificar, fijar seguimiento, cerrar la entrevista). | 8,1 | 9,0 | 1,0 | 9,1 |
| 6. Identificar los aspectos de la comunicación médico-paciente que en estudios científicos se han mostrado eficientes (por relacionarse positivamente con resultados de la asistencia). | 8,0 | 8,0 | 2,0 | 9,1 |
| 7. Reconocer los mecanismos a través de los cuales la comunicación clínica conlleva una mejora de resultados de la asistencia generalmente a través de resultados intermedios. | 7,5 | 8,0 | 1,0 | 13,6 |
| 8. Realizar una entrevista médica integrando sus contenidos (anamnesis, exploración, diagnóstico, plan terapéutico y evolución) con el proceso (habilidades comunicacionales o de relación). | 8,7 | 9,0 | 0,0 | 2,3 |
| 9. Mostrar que acepta la importancia del contexto relacional en el que se desarrolla la entrevista clínica empleando conductas adecuadas para su consideración. | 8,1 | 9,0 | 1,5 | 9,1 |
| 10. Mostrar disposición para involucrar al paciente en la interacción, estableciendo una relación terapéutica usando una aproximación centrada en el paciente. | 8,6 | 9,0 | 1,0 | 2,3 |

| **A.2. Tareas y Habilidades para comunicar con los pacientes** | | | | |
| --- | --- | --- | --- | --- |
| **A.2.1. Establecer y mantener una relación terapéutica (Conectar)** (El estudiante establece y mantiene una relación terapéutica mediante una aproximación centrada en el paciente) **El estudiante será capaz de…** | **X*** | **Me*** | **RI*** | **% fuera Me*** |
| 11. Conocer los aspectos más relevantes de la comunicación no verbal (contacto visual-facial, gestos, expresiones faciales, proxémica, paralenguaje…) y su influencia en el establecimiento de una relación efectiva. | 8,4 | 9,0 | 1,0 | 6,8 |
| 12. Comprobar que el paciente se siente atendido y escuchado, mediante técnicas tales como escucha activa, preguntas, comprobaciones, etc. | 8,8 | 9,0 | 0,0 | 0,0 |
| 13. Percibir el lenguaje no verbal del paciente y responder de forma adecuada al contexto. | 8,6 | 9,0 | 1,0 | 2,3 |
| 14. Utilizar los registros de historia clínica (manual/informatizados) en la comunicación con el paciente de forma que no la interfieran. | 8,1 | 9,0 | 1,0 | 6,8 |
| 15. Aplicar habilidades sociales comunicacionales para recibir a los pacientes que fomenten el mantenimiento de una relación efectiva (saludar, llamar por su nombre al paciente, acomodarlo…). | 8,7 | 9,0 | 0,0 | 2,3 |
| 16. Aplicar habilidades sociales comunicacionales para despedir a los pacientes que fomenten el mantenimiento de una relación efectiva (despedir, acompañar…). | 8,7 | 9,0 | 0,0 | 2,3 |
| 17. Mostrar empatía en los momentos oportunos (aparición de emociones, situaciones difíciles…). | 8,6 | 9,0 | 0,5 | 2,3 |
| 18. Reconocer situaciones difíciles y desafíos comunicacionales (llanto, emociones fuertes, interrupciones, agresiones, enfado, ansiedad, temas sensibles o embarazosos, dificultades cognitivas, malas noticias, primer encuentro…). | 8,6 | 9,0 | 1,0 | 4,6 |
| 19. Usar técnicas para afrontar con sensibilidad y de forma constructiva situaciones difíciles y desafíos comunicacionales. | 8,4 | 9,0 | 1,0 | 4,6 |
| 20. Relacionarse con el paciente de manera respetuosa teniendo en cuenta sus derechos (confidencialidad, privacidad, autonomía, respeto a valores y creencias). | 8,8 | 9,0 | 0,0 | 2,3 |
| 21. Considerar al paciente como un colaborador para construir la relación y tratarlo como tal. | 7,8 | 9,0 | 1,5 | 13,6 |
| 22. Mostrar interés genuino en la relación con el paciente y su situación. | 8,1 | 9,0 | 1,5 | 9,1 |
| 23. Utilizar de manera adecuada el sentido del humor en la relación con el paciente (en situaciones que requieran distensión del ambiente, para el acercamiento…). | 7,2 | 8,0 | 3,0 | 27,3 |

| **A.2.2. Intercambiar Información y Comprenderla** | | | | |
| --- | --- | --- | --- | --- |
| **A.2.2.1. Obtener la información** (El estudiante recoge la información relevante para el razonamiento y la toma de decisiones clínicas) **El estudiante será capaz de…** | **X*** | **Me*** | **RI*** | **% fuera Me*** |
| 24. Diferenciar dolencia (illness) y enfermedad (disease), reconociendo la importancia de explorar ambas perspectivas. | 8,4 | 9,0 | 1,0 | 2,3 |
| 25. Reconocer las ventajas e inconvenientes de las diferentes habilidades comunicacionales para obtener información (preguntas abiertas/cerradas, facilitación…). | 8,2 | 9,0 | 1,5 | 6,8 |
| 26. Delimitar de manera precisa el/los motivo/s de consulta del paciente (pregunta abierta, sin interrumpir, explorando diferentes motivos…). | 8,4 | 9,0 | 1,0 | 4,6 |
| 27. Explorar y obtener el contenido de la historia bio-psico-social del paciente (somático, mental, psicológico, familiar, laboral) cuando la situación lo requiera. | 8,3 | 9,0 | 1,0 | 6,8 |
| 28. Añadir a la historia clínica cualquier otro elemento de interés desde una perspectiva de la Medicina Centrada en la Persona (necesidades espirituales, dificultades económicas, interferencias con el ocio…) que habitualmente no se registran en los formatos de historia. | 8,2 | 8,5 | 1,0 | 6,8 |
| 29. Utilizar diferentes tipos de preguntas (abiertas, cerradas y dirigidas…) adecuadas a cada situación. | 8,1 | 9,0 | 1,0 | 9,1 |
| 30. Utilizar técnicas verbales y no verbales de escucha activa (reflexión, captar pistas del paciente, parafrasear, facilitar, resumir…). | 8,6 | 9,0 | 1,0 | 0,0 |
| 31. Resumir al paciente la información obtenida como forma de comprobación. | 8,1 | 9,0 | 2,0 | 13,6 |
| 32. Valorar como afecta al paciente su dolencia en la vida diaria, entorno sociofamiliar o laboral. | 8,5 | 9,0 | 1,0 | 4,6 |
| 33. Considerar otros factores que puedan influir en las necesidades del paciente cuando consulta (ideas, temores, sentimientos, preferencias, experiencias previas…). | 8,5 | 9,0 | 1,0 | 2,3 |
| 34. Establecer un acompañamiento adecuado de la exploración física (pidiendo permiso, explicando lo que se va a hacer y por qué, compartiendo los hallazgos con el paciente…). | 8,5 | 9,0 | 1,0 | 2,3 |
| 35. Reconocer las divergencias entre los valores y normas del médico y los del paciente, respetándolos sin juzgarlos. | 8,4 | 9,0 | 1,0 | 2,3 |
| 36. Mostrar apertura y disposición para tratar de manera apropiada cualquier aspecto importante para el paciente en relación con su salud empleando conductas adecuadas para su consideración. | 7,9 | 8,0 | 2,0 | 6,8 |

| **A.2.2.2. Ofrecer la información** (El estudiante ofrece de forma clara y personalizada la información que el paciente necesita para tomar decisiones) **El estudiante será capaz de…** | **X*** | **Me*** | **RI*** | **% fuera Me*** |
| --- | --- | --- | --- | --- |
| 37. Valorar críticamente los resultados de la investigación científica sobre la transmisión de información a los pacientes y sus implicaciones en la práctica clínica. | 7,9 | 8,0 | 2,0 | 11,4 |
| 38. Describir los principios básicos para informar sobre riesgos de forma adecuada (evitar las manipulaciones de cualquier tipo y/o parcialidad en la presentación de cifras y probabilidades…). | 8,3 | 9,0 | 1,0 | 6,8 |
| 39. Comunicar el riesgo al paciente haciendo un uso personalizado de los indicadores (medidas de riesgo). | 7,7 | 8,0 | 2,0 | 18,2 |
| 40. Suplementar esta información verbal con diagramas, modelos, información escrita e instrucciones cuando sea necesario. | 8,1 | 8,5 | 1,0 | 11,4 |
| 41. Estimar el nivel de conocimiento del paciente sobre su problema y hasta dónde desea saber, para entregar la información que realmente requiere. | 8,5 | 9,0 | 1,0 | 2,3 |
| 42. Ofrecer la información al paciente de forma oportuna (circunstancia adecuada). | 8,3 | 9,0 | 1,0 | 6,8 |
| 43. Adaptar la comunicación al nivel de comprensión y lenguaje del paciente, evitando jerga médica. | 8,7 | 9,0 | 0,0 | 0,0 |
| 44. Proporcionar información centrada en el paciente, incorporando su perspectiva y haciéndola significativa para él. | 8,4 | 9,0 | 1,0 | 4,6 |
| 45. Discutir de manera centrada en el paciente, beneficios, riesgos y resultados esperados. | 8,1 | 9,0 | 1,0 | 9,1 |
| 46. Comprobar que el paciente ha entendido la información suministrada, facilitando la expresión de dudas. | 8,6 | 9,0 | 0,0 | 2,3 |
| 47. Explicar al paciente la información precisa para minimizar la incertidumbre en el proceso de toma de decisiones. | 8,1 | 9,0 | 1,0 | 6,8 |
| 48. Compartir, con el consentimiento del paciente, la información con terceros (colegas, familia y otros…). | 8,0 | 9,0 | 2,0 | 15,9 |

| **A.2.3. Acordar y Ayudar al paciente a llevar a cabo lo acordado en la tomar de decisiones** (El estudiante toma decisiones considerando la participación y responsabilidad del paciente, tomando en consideración sus preferencias) **El estudiante será capaz de…** | **X*** | **Me*** | **RI*** | **% fuera Me*** |
| --- | --- | --- | --- | --- |
| 49. Diferenciar los distintos modelos de participación del paciente en la toma de decisiones (paternalista, consumista, colaborador…). | 7,3 | 8,0 | 2,5 | 25,0 |
| 50. Determinar cuál debe ser su rol como médico más oportuno en el proceso de toma de decisiones ante cada paciente. | 7,8 | 8,0 | 2,0 | 13,6 |
| 51. Aceptar el papel de la incertidumbre como elemento sustancial del razonamiento clínico y de la toma de decisiones. | 7,9 | 9,0 | 2,0 | 13,6 |
| 52. Reconocer los elementos que contribuyen a la presencia de incertidumbre (falta de conocimiento del profesional, ausencia de evidencia…) en el ámbito de la toma de decisiones clínicas. | 7,6 | 8,5 | 2,0 | 20,5 |
| 53. Comunicar a los pacientes que existe incertidumbre de manera adaptada al grado de tolerancia de éste. | 7,8 | 8,0 | 2,0 | 22,7 |
| 54. Explorar las necesidades, recursos (información, autonomía, confianza, responsabilidad, rasgos psicológicos…) y disposición del paciente para facilitar su implicación en la toma de decisiones. | 8,3 | 9,0 | 1,0 | 4,6 |
| 55. Alcanzar acuerdos con el paciente utilizando habilidades de negociación. | 8,0 | 9,0 | 1,5 | 6,8 |
| 56. Comprender el papel en la práctica clínica de las ayudas para la toma de decisiones (decision aids). | 6,9 | 7,5 | 2,0 | 29,6 |
| 57. Capacitar al paciente en el uso de ayudas para la toma de decisiones para utilizarlas en la discusión. | 6,9 | 8,0 | 3,0 | 29,6 |
| 58. Clarificar con el paciente cómo y cuándo debe tomarse la decisión. | 7,7 | 8,0 | 2,0 | 15,9 |
| 59. Discutir con el paciente el espectro de las posibles consecuencias de una decisión (explicarle las consecuencias de escoger/no escoger la opción discutida). | 8,0 | 8,0 | 1,0 | 6,8 |
| 60. Ofrecer al paciente la opción de abrir y enriquecer la discusión de toma de decisiones incluyendo a terceros (colegas, familiares). | 7,9 | 8,0 | 2,0 | 11,4 |
| 61. Usar el consentimiento informado de forma que el paciente comprenda las características y consecuencias del procedimiento. | 8,3 | 9,0 | 1,0 | 6,8 |
| 62. Adaptar el plan / intervención a los recursos y fortalezas del paciente. | 8,5 | 9,0 | 1,0 | 0,0 |
| 63. Cerrar el proceso al final de la consulta utilizando las estrategias comunicacionales adecuadas (resumiendo, resaltando aspectos claves, anticipando posibles evoluciones y orientando al respecto…). | 8,7 | 9,0 | 0,5 | 0,0 |
| 64. Asumir la implicación del paciente y su responsabilidad en el proceso de toma de decisiones y emplear conductas adecuadas para ello. | 7,8 | 8,0 | 2,0 | 20,5 |
| 65. Estar dispuesto a reevaluar y revisar sus propias decisiones. | 8,2 | 9,0 | 1,0 | 9,1 |

| **B) COMUNICACION CON LA FAMILIA DEL PACIENTE**  **B.1. Contexto familiar del paciente** (El estudiante reconoce y evalúa el papel de la familia en la atención clínica a pacientes y establece una comunicación efectiva con ella en beneficio de éste) **El estudiante será capaz de…** | **X** | **Me** | **RI** | **% fuera Me** |
| --- | --- | --- | --- | --- |
| 66. Conocer el papel de la familia como un sistema en la atención y cuidados de los pacientes. | 8,2 | 9,0 | 1,5 | 9,1 |
| 67. Describir los modelos básicos que explican el comportamiento de las familias y del paciente como uno de sus miembros (el ciclo vital individual y familiar). | 7,6 | 8,0 | 2,0 | 20,5 |
| 68. Conocer y emplear los métodos y herramientas para la identificación de la estructura y funcionamiento familiar (genogramas o familiograma, función familiar, acontecimientos estresantes, red social…). | 7,2 | 7,0 | 3,0 | 27,3 |
| 69. Considerar los patrones de respuesta de la familia ante la enfermedad y los acontecimientos vitales estresantes cuando se atiende a un paciente. | 7,6 | 8,0 | 2,0 | 15,9 |
| 70. Identificar al miembro(s) de la familia que cumple el rol de cuidador principal, para incorporarlo al proceso y valorarlo en este rol. | 7,9 | 9,0 | 1,0 | 11,4 |
| 71. Solicitar y sintetizar la información relevante de otros miembros de la familia y cuidadores del paciente, si es necesario y se encuentran disponibles. | 8,0 | 8,0 | 1,5 | 9,1 |
| 72. Establecer una comunicación efectiva con el paciente y su familia para la identificación de los problemas, detección de los recursos y puesta en marcha de los planes de actuación en beneficio del paciente. | 8,4 | 9,0 | 1,0 | 2,3 |
| 73. Ayudar a la familia a tomar decisiones cuando el paciente es menor de edad o se encuentra incapacitado (demencias, pacientes en coma, problemas mentales incapacitantes…). | 8,5 | 9,0 | 1,0 | 2,3 |
| 74. Reconocer retos comunicacionales específicos con familiares (la confidencialidad, el secretismo, el acompañante enfermo… | 8,4 | 9,0 | 1,0 | 0,0 |
| 75. Mostrar disposición para incorporar a la familia y trabajar con ella en beneficio del paciente. | 8,3 | 9,0 | 1,5 | 4,6 |
| 76. Mostrar disposición a facilitar la comunicación entre los miembros de la familia del paciente empleando conductas adecuadas para ello. | 7,6 | 8,5 | 2,0 | 13,6 |
| 77. Mostrar sensibilidad con los miedos y preocupaciones de los familiares empleando conductas adecuadas para ello. | 8,3 | 9,0 | 1,0 | 2,3 |

| **C) COMUNICACIÓN INTRAPERSONAL (AUTOPERCEPCIÓN)** **C.1. El médico como persona** (auto-conocimiento, auto-reflexión, auto-crítica y auto-cuidados) (El estudiante habitualmente reflexiona sobre su comportamiento y la forma en la que comunica, desarrollando y mejorando su auto-conocimiento, auto-reflexión, auto-crítica, auto-cuidados)  **El estudiante será capaz de…** | **X** | **Me** | **RI** | **% fuera Me** |
| --- | --- | --- | --- | --- |
| 78. Describir los factores que influyen en la relación entre el profesional y el paciente (estereotipos, prejuicios socioculturales, experiencias, intereses…). | 7,9 | 8,5 | 2,0 | 15,9 |
| 79. Reflexionar críticamente su propio estilo comunicativo y conductual considerando posibles alternativas. | 8,3 | 9,0 | 1,0 | 4,6 |
| 80. Reconocer las barreras que dificultan el autoconomiento, y utilizar técnicas y estrategias para su promoción tales como práctica reflexiva, perspectiva personal … | 8,3 | 9,0 | 1,0 | 6,8 |
| 81. Identificar las señales de sobrecarga de trabajo y estrés (insomnio, ansiedad, alteraciones del sueño…). | 8,3 | 9,0 | 1,0 | 4,6 |
| 82. Distinguir las principales fuentes de errores médicos (deficiente información o evaluación de las necesidades del paciente, inadecuada comunicación…). | 8,1 | 9,0 | 1,5 | 4,6 |
| 83. Reconocer los errores técnicos, sesgos cognitivos y reacciones emocionales que dificultan el desarrollo de relaciones terapéuticas. | 8,2 | 9,0 | 1,0 | 4,6 |
| 84. Utilizar estrategias para reducir el estrés y la sobrecarga (de relajación, grupos de reflexión, grupos Balint, supervisión y apoyo…). | 7,8 | 9,0 | 2,0 | 18,2 |
| 85. Controlar las propias reacciones emocionales y trabajar de forma eficiente, aún en situaciones difíciles (alto grado de sufrimiento del paciente, paciente demandante…). | 8,0 | 9,0 | 1,0 | 11,4 |
| 86. Desarrollar los hábitos mentales necesarios para el reconocimiento de sesgos propios, mediante el uso de técnicas específicas (preguntas reflexivas, observación con perspectiva, presencia plena (*mindfulness*), suspensión del juicio, actitud no prejuiciosa…). | 8,0 | 8,0 | 2,0 | 6,8 |
| 87. Reconocer errores propios (y ajenos), asumirlos como parte constitutiva del trabajo y buscar soluciones a los mismos (asistencia de superiores…). | 8,5 | 9,0 | 1,0 | 0,0 |
| 88. Reconocer sus propias emociones (inseguridad, simpatía/antipatía, atracción…) en relación a los otros (pacientes, colegas). | 8,1 | 9,0 | 1,0 | 6,8 |
| 89. Valorar las fortalezas y debilidades personales determinantes de su autopercepción en los contextos docentes adecuados (tutorías, mentorías, grupos de reflexión…). | 8,2 | 8,5 | 1,0 | 6,8 |
| 90. Aceptar y abordar la incertidumbre propia de manera adecuada al estadío educativo | 7,7 | 8,0 | 2,0 | 13,6 |

| **D) COMUNICACIÓN INTER-INTRA PROFESIONAL** **D.1. El contexto profesional del médico: Comunicación inter e intra profesional** (El estudiante se comunica eficientemente con los profesionales que forman parte de su equipo o fuera de este) **El estudiante será capaz de…** | **X** | **Me** | **RI** | **% fuera Me** |
| --- | --- | --- | --- | --- |
| 91. Identificar los principios básicos de la dinámica de grupo así como de sus factores favorecedores e inhibidores. | 7,7 | 8,0 | 2,0 | 15,9 |
| 92. Identificar los diferentes miembros de los distintos equipos de salud interprofesionales y sus respectivas responsabilidades. | 8,0 | 9,0 | 1,0 | 9,1 |
| 93. Clarificar su propio rol y responsabilidades como estudiante en los equipos profesionales con los que interactúe. | 8,1 | 9,0 | 1,0 | 11,4 |
| 94. Identificar cuando derivar a profesionales/ instituciones/agencias que pueden ayudar a solventar los problemas en función de cada situación. | 8,2 | 9,0 | 1,5 | 6,8 |
| 95. Describir los principios y estrategias para negociar y resolver conflictos con otros profesionales y usarlos adecuadamente. | 7,5 | 8,0 | 2,0 | 22,7 |
| 96. Discutir decisiones de forma apropiada con colegas, pacientes y sus familiares, y si es necesario, reevaluar sus propias decisiones. | 8,2 | 9,0 | 1,0 | 9,1 |
| 97.  Asegurar que toda la información clínica relevante del paciente está disponible. | 7,4 | 8,5 | 2,0 | 18,2 |
| 98.  Facilitar el flujo de información de las opiniones en el grupo y promover que los miembros del equipo den opiniones divergentes. | 7,9 | 9,0 | 2,0 | 13,6 |
| 99. Dar feedback a los miembros del equipo de forma apropiada (comentarios en primera persona, resaltar primero lo positivo, no juzgar). | 8,0 | 9,0 | 2,0 | 9,1 |
| 100. Contribuir de manera efectiva a la continuidad de la atención en la referencia/derivación y retorno de los pacientes entre los diferentes niveles asistenciales (primaria, especializada). | 7,8 | 8,5 | 2,0 | 13,6 |
| 101. Hacer presentaciones clínicas o científicas en público de forma efectiva. | 7,6 | 8,0 | 2,0 | 15,9 |
| 102. Dar instrucciones de forma clara y precisa. | 8,3 | 9,0 | 1,0 | 9,1 |
| 103. Contribuir a crear una atmósfera de trabajo positiva (apoyar e integrar a los diferentes miembros del equipo, mencionar el lado positivo de los aspectos desagradables, valorar el éxito del equipo…). | 8,4 | 9,0 | 1,0 | 6,8 |
| 104. Respetar la individualidad, la percepción subjetiva de los miembros del equipo y la maestría (pericia) de los diferentes profesionales de salud. | 8,4 | 9,0 | 1,0 | 6,8 |
| 105. Mantener la confidencialidad sobre las decisiones tomadas en el equipo. | 8,5 | 9,0 | 1,0 | 6,8 |
| 106.  Ser asertivo con el resto de miembros del equipo. | 7,8 | 8,0 | 2,0 | 13,6 |
| 107. Mostar actitud negociadora para alcanzar acuerdos empleando conductas adecuadas para ello. | 8,2 | 8,0 | 2,0 | 4,6 |
| 108. Mostar flexibilidad para cambiar su rol dentro de un equipo de trabajo. | 8,3 | 9,0 | 1,0 | 4,6 |

| **E) COMUNICACIÓN POR DIFERENTES VIAS E.1. Canales comunicacionales (El estudiante utiliza con eficiencia diferentes formas de comunicarse)** | | | | |
| --- | --- | --- | --- | --- |
| **E.1.1. Comunicación directa (cara a cara)** **El estudiante será capaz de…** | **X** | **Me** | **RI** | **% fuera Me** |
| 109. Identificar los factores ambientales (físicos y sociales) que pueden obstaculizar la comunicación interpersonal en los diferentes contextos. | 8,3 | 9,0 | 1,0 | 4,5 |
| 110. Identificar si existe discrepancia entre los componentes verbales y no verbales de la comunicación. | 8,2 | 9,0 | 1,5 | 9,1 |
| 111. Emplear adecuadamente la proxémica (distancia física de comunicación). | 8,2 | 9,0 | 1,0 | 6,8 |
| **E.1.2. Comunicación escrita El estudiante será capaz de…** | **X** | **Me** | **RI** | **% fuera Me** |
| 112. Reconocer los formatos y soportes de historia clínica y los documentos habitualmente utilizados para la comunicación escrita con pacientes y entre profesionales (informes de alta, derivación, para solicitud de pruebas,…). | 8,1 | 9,0 | 1,5 | 13,6 |
| 113. Registrar la valoración inicial de un paciente así como la posterior evolución clínica diaria en un lenguaje escrito conciso y claro. | 8,1 | 9,0 | 1,0 | 13,6 |
| 114. Escribir un informe de alta y de derivación, de forma estructurada, comprensible, suficiente y clara. | 8,2 | 9,0 | 1,0 | 11,4 |
| 115. Escribir peticiones de pruebas y prescripciones de forma precisa, clara y justificada. | 8,0 | 9,0 | 2,0 | 11,4 |
| 116. Mantener unos registros claros, apropiados, sobre la información relevante del encuentro clínico. | 8,2 | 9,0 | 1,0 | 9,1 |
| 117. Escribir documentos legales habituales (certificados de defunción, certificados de salud…). | 8,3 | 9,0 | 1,0 | 6,8 |
| **E.1.3. Comunicación informática o computacional El estudiante será capaz de…** | **X** | **Me** | **RI** | **% fuera Me** |
| 118. Reconocer las diferentes tecnologías de la información más utilizadas en el ámbito sanitario. | 7,7 | 8,0 | 2,0 | 15,9 |
| 119. Conocer los registros electrónicos de los pacientes así como con los sistemas de prescripción y derivación. | 8,0 | 8,0 | 2,0 | 6,8 |
| 120. Manejar las tecnologías de la información (e-mails, WhatsApp, web2.0,…) en aspectos de atención sanitaria garantizando la confidencialidad. | 7,9 | 8,0 | 2,0 | 15,9 |
| **E.1.4. Comunicación telefónica El estudiante será capaz de…** | **X** | **Me** | **RI** | **% fuera Me** |
| 121. Reconocer los usos y las limitaciones de la comunicación telefónica con pacientes. | 8,0 | 9,0 | 2,0 | 11,4 |
| 122. Comunicar telefónicamente con pacientes atendiendo a las demandas específicas y adaptaciones comunicacionales que este medio requiere. | 7,8 | 8,0 | 2,0 | 13,6 |

| **F) COMUNICACIÓN EN SITUACIONES ESPECIALES F.1. Contextos comunicacionales específicos (El estudiante aplica y adapta las habilidades comunicacionales nucleares ante situaciones clínicas específicas y utiliza habilidades concretas que cada situación pueda requerir)** | | | | |
| --- | --- | --- | --- | --- |
| **F.1.1. Situaciones sensibles El estudiante será capaz de…** | **X** | **Me** | **RI** | **% fuera Me** |
| 123. Reconocer situaciones delicadas que representen retos comunicacionales (del tipo de dar malas noticias, afrontar temas sobre el final de la vida, situaciones de duelo, historia sexual, violencia de género, maltrato infantil, infección VIH, explicar situaciones de incertidumbre clínica…). | 8,5 | 9,0 | 1,0 | 4,5 |
| 124. Abordar algunas de ellas de manera sensible y constructiva mediante la aplicación y adaptación de las habilidades comunicacionales nucleares y la utilización de estrategias y habilidades específicas que cada una de ellas pueda requerir. | 8,2 | 9,0 | 1,0 | 9,1 |
| 125. Conocer los aspectos legales imprescindibles vigentes en cada legislación relacionados con el manejo de algunas de estas situaciones. | 7,9 | 9,0 | 2,0 | 13,6 |
| **F.1.2. Manejo de las emociones El estudiante será capaz de…** | **X** | **Me** | **RI** | **% fuera Me** |
| 126. Reconocer situaciones de tensión emocional en las consultas (del tipo de estrés, temor, enfado, agresividad, negación, colusión, vergüenza…). | 8,3 | 9,0 | 1,0 | 4,5 |
| 127. Abordar algunas de ellas de manera sensible y constructiva mediante la aplicación y adaptación de las habilidades comunicacionales nucleares y la utilización de estrategias y habilidades específicas que cada una de ellas pueda requerir. | 7,9 | 8,5 | 1,5 | 9,1 |
| **F.1.3. Diversidad cultural y social El estudiante será capaz de…** | **X** | **Me** | **RI** | **% fuera Me** |
| 128. Reconocer la diversidad cultural y social de los pacientes (etnicidad, nacionalidad, estatus socioeconómico, idioma, religión, género, valores, sexualidad…) y las dificultades comunicacionales que esto conlleva. | 8,4 | 9,0 | 1,0 | 6,8 |
| 129. Abordar algunas de ellas de manera sensible y constructiva mediante la aplicación y adaptación de las habilidades comunicacionales nucleares y la utilización de estrategias y habilidades específicas que cada una de ellas pueda requerir. | 8,0 | 8,5 | 1,0 | 13,6 |
| **F.1.4. Promoción de la salud y modificación de conductas El estudiante será capaz de…** | **X** | **Me** | **RI** | **% fuera Me** |
| 130. Describir los principios básicos de la motivación. | 7,7 | 8,5 | 2,0 | 13,6 |
| 131. Reconocer los estadios del proceso de cambio en el que se encuentra un paciente a la hora de modificar conductas o seguir tratamientos. | 8,0 | 8,5 | 2,0 | 9,1 |

| 132. Explorar el grado de motivación del paciente para realizar cambios. | 8,2 | 9,0 | 1,5 | 6,8 |
| --- | --- | --- | --- | --- |
| 133. Aplicar estrategias comunicacionales eficaces para la modificación de conductas. | 8,1 | 9,0 | 1,0 | 11,4 |
| 134. Asumir un enfoque preventivo y de promoción de la salud en la atención a pacientes y emplear conductas adecuadas para ello. | 8,4 | 9,0 | 1,0 | 2,3 |
| **F.1.5. Contextos clínicos específicos El estudiante será capaz de…** | **X** | **Me** | **RI** | **% fuera Me** |
| 135. Abordar algunos contextos clínicos específicos (psiquiátricos, pacientes con demencia, con problemas sensoriales: auditivos, visuales, de expresión verbal) mediante la aplicación y adaptación de las habilidades comunicacionales nucleares y la utilización de estrategias y habilidades específicas que cada una de ellas pueda requerir. | 8,1 | 9,0 | 2,0 | 11,4 |
| **F.1.6. Pacientes de diferentes edades El estudiante será capaz de…** | **X** | **Me** | **RI** | **% fuera Me** |
| 136. Comunicar con pacientes de diferentes grupos etareos (niños y padres, adolescentes, ancianos) mediante la aplicación y adaptación de las habilidades comunicacionales nucleares y la utilización de estrategias y habilidades específicas que cada uno de ellos pueda requerir. | 8,3 | 9,0 | 1,5 | 2,3 |

***X:** Media

***Me:** Mediana

***RI:** Rango intercuartílico

***% fuera de Me:** Porcentaje de encuestados fuera de la región que incluye la mediana
