# Supplementary material for: A Latin American, Portuguese and Spanish consensus on a core communication curriculum for undergraduate medical education
Source: BMC Med Educ. 2016 Mar 28;16:99. doi: 10.1186/s12909-016-0610-8 (PMC4809037; doi:10.1186/s12909-016-0610-8)
Supplement: Additional file 3: — LAPS_CCC Portuguese version. (DOCX 36 kb) [file 12909_2016_610_MOESM3_ESM.docx]

| 1. **COMUNICAÇÃO COM O PACIENTE (DÍADE)**   **A.1. Aspetos gerais da Entrevista Clínica com pacientes** (O estudante reconhece o valor da entrevista para o objetivo do ato clínico conhecendo, integrando e estruturando os seus diferentes componentes)  **O estudante será capaz de…** | **X*** | **Me*** | **RI*** | **% fuera Me*** |
| --- | --- | --- | --- | --- |
| 1. Explicar os princípios e as características da comunicação humana. | 7,4 | 7,5 | 2,0 | 13,6 |
| 1. Explicar os modelos de relação entre profissional e paciente (centrados no profissional, no paciente, em tarefas, no processo, mistos…). | 8,0 | 8,0 | 2,0 | 2,3 |
| 1. Descrever os diferentes elementos do conteúdo de uma história clínica (anamnese: sentir-se doente (illness) e doença (disease), exploração física e complementar, aproximação diagnóstica, plano terapêutico, evolução). | 8,1 | 9,0 | 1,0 | 6,8 |
| 1. Descrever os diferentes elementos de processo úteis para a elaboração de uma história clínica (aptidões comunicacionais ou de relação). | 7,7 | 8,0 | 2,0 | 13,6 |
| 1. Delimitar a estrutura de uma entrevista clínica desde o seu início até ao seu final (introdução, iniciar a entrevista, partilhar informação: obtê-la e oferecê-la, planificar, fixar seguimento, fechar a entrevista). | 8,1 | 9,0 | 1,0 | 9,1 |
| 1. Identificar os aspetos da comunicação médico-paciente que em estudos científicos se mostraram eficientes (por se relacionarem positivamente com os resultados da assistência). | 8,0 | 8,0 | 2,0 | 9,1 |
| 1. Reconhecer os mecanismos através dos quais a comunicação clínica implica uma melhoria de resultados da assistência, geralmente através de resultados intermédios. | 7,5 | 8,0 | 1,0 | 13,6 |
| 1. Realizar uma entrevista médica integrando os seus conteúdos (anamnese, exploração, diagnóstico, plano terapêutico e evolução) com o processo (habilidades comunicacionais ou de relação). | 8,7 | 9,0 | 0,0 | 2,3 |
| 1. Mostrar que aceita a importância do contexto relacional em que se desenvolve a entrevista clínica utilizando condutas adequadas para a sua consideração. | 8,1 | 9,0 | 1,5 | 9,1 |
| 1. Mostrar disposição para envolver o paciente na interação, estabelecendo uma relação terapêutica usando uma aproximação centrada no paciente. | 8,6 | 9,0 | 1,0 | 2,3 |

| **A.2. Tarefas e Aptidões para comunicar com os pacientes** | | | | |
| --- | --- | --- | --- | --- |
| **A.2.1. Estabelecer e manter uma relação terapêutica (Conectar)** (O estudante estabelece e mantem uma relação terapêutica mediante uma aproximação centrada no paciente)  **O estudante será capaz de…** | **X*** | **Me*** | **RI*** | **% fuera Me*** |
| 1. Conhecer os aspetos mais relevantes da comunicação não-verbal (contacto visual-facial, gestos, expressões faciais, proxémica, paralinguagem…) e a sua influência no estabelecimento de uma relação efetiva. | 8,4 | 9,0 | 1,0 | 6,8 |
| 1. Comprovar que o paciente se sente atendido e ouvido, mediante técnicas tais como escuta ativa, perguntas, comprovações, etc. | 8,8 | 9,0 | 0,0 | 0,0 |
| 1. Perceber a linguagem não-verbal do paciente e responder de forma adequada ao contexto. | 8,6 | 9,0 | 1,0 | 2,3 |
| 1. Utilizar os registos da história clínica (manual/informatizados) na comunicação com o paciente de forma que não interfiram. | 8,1 | 9,0 | 1,0 | 6,8 |
| 1. Aplicar habilidades sociais e comunicacionais para receber os pacientes as quais fomentam a manutenção de uma relação efetiva (cumprimentar, chamar o paciente pelo seu nome, acomodá-lo…). | 8,7 | 9,0 | 0,0 | 2,3 |
| 1. Aplicar habilidades sociais comunicacionais para se despedir dos pacientes as quais fomentam a manutenção de uma relação efetiva (despedir-se, acompanhar…). | 8,7 | 9,0 | 0,0 | 2,3 |
| 1. Mostrar empatia nos momentos oportunos (aparecimento de emoções, situações difíceis…). | 8,6 | 9,0 | 0,5 | 2,3 |
| 1. Reconhecer situações difíceis e desafios comunicacionais (choro, emoções fortes, interrupções, agressões, irritação, ansiedade, temas sensíveis ou incómodos, dificuldades cognitivas, más notícias, primeiro encontro…). | 8,6 | 9,0 | 1,0 | 4,6 |
| 1. Usar técnicas para afrontar com sensibilidade e de forma construtiva situações difíceis e desafios comunicacionais. | 8,4 | 9,0 | 1,0 | 4,6 |
| 1. Relacionar-se com o paciente de maneira respeitosa tendo em conta os seus direitos (confidencialidade, privacidade, autonomia, respeito pelos valores e crenças). | 8,8 | 9,0 | 0,0 | 2,3 |
| 1. Considerar o paciente como um colaborador para construir a relação e tratá-lo como tal. | 7,8 | 9,0 | 1,5 | 13,6 |
| 1. Mostrar interesse genuíno na relação com o paciente e na sua situação. | 8,1 | 9,0 | 1,5 | 9,1 |
| 1. Utilizar de maneira adequada o sentido do humor na relação com o paciente (em situações que requerem distensão do ambiente, para aproximação…). | 7,2 | 8,0 | 3,0 | 27,3 |

| **A.2.2. Trocar Informação e Compreendê-la** | | | | |
| --- | --- | --- | --- | --- |
| **A.2.2.1. Obter a informação** (O estudante recolhe a informação relevante para o raciocínio e a tomada de decisões clínicas)  **O estudante será capaz de…** | **X*** | **Me*** | **RI*** | **% fuera Me*** |
| 1. Diferenciar sentir-se doente (illness) e doença (disease), reconhecendo a importância de explorar ambas perspetivas. | 8,4 | 9,0 | 1,0 | 2,3 |
| 1. Reconhecer as vantagens e os inconvenientes das diferentes habilidades comunicacionais para obter informação (perguntas abertas/fechadas, facilitação…). | 8,2 | 9,0 | 1,5 | 6,8 |
| 1. Delimitar de maneira precisa o/os motivo/s da consulta do paciente (pergunta aberta, sem interromper, explorando diferentes motivos…). | 8,4 | 9,0 | 1,0 | 4,6 |
| 1. Explorar e obter o conteúdo da história bio-psico-social do paciente (somático, mental, psicológico, familiar, laboral) quando a situação o requer. | 8,3 | 9,0 | 1,0 | 6,8 |
| 1. Acrescentar à história clínica qualquer outro elemento de interesse desde uma perspetiva da Medicina Centrada na Pessoa (necessidades espirituais, dificuldades económicas, interferências com o lazer…) que habitualmente não se registam nos formatos da história. | 8,2 | 8,5 | 1,0 | 6,8 |
| 1. Utilizar diferentes tipos de perguntas (abertas, fechadas e dirigidas…) adequadas a cada situação. | 8,1 | 9,0 | 1,0 | 9,1 |
| 1. Utilizar técnicas verbais e não-verbais de escuta ativa (reflexão, captar pistas do paciente, parafrasear, facilitar, resumir…). | 8,6 | 9,0 | 1,0 | 0,0 |
| 1. Resumir ao paciente a informação obtida como forma de comprovação. | 8,1 | 9,0 | 2,0 | 13,6 |
| 1. Valorizar como afeta ao paciente a sua dolência na vida diária, ambiente sociofamiliar ou laboral. | 8,5 | 9,0 | 1,0 | 4,6 |
| 1. Considerar outros fatores que possam influenciar as necessidades do paciente durante a consulta (ideias, medos, sentimentos, preferências, experiências prévias…). | 8,5 | 9,0 | 1,0 | 2,3 |
| 1. Estabelecer um acompanhamento adequado da exploração física (pedindo autorização, explicando o que se vai fazer e porquê, partilhando as descobertas com o paciente…). | 8,5 | 9,0 | 1,0 | 2,3 |
| 1. Reconhecer as divergências entre os valores e as normas do médico e os do paciente, respeitando-os sem julgar. | 8,4 | 9,0 | 1,0 | 2,3 |
| 1. Mostrar abertura e disposição para tratar de maneira apropriada qualquer aspeto importante para o paciente relativo à sua saúde e empregando condutas adequadas para a sua consideração. | 7,9 | 8,0 | 2,0 | 6,8 |

| **A.2.2.2. Oferecer a informação** (O estudante oferece de forma clara e personalizada a informação que o paciente necessita para tomar decisões)  **O estudante será capaz de…** | **X*** | **Me*** | **RI*** | **% fuera Me*** |
| --- | --- | --- | --- | --- |
| 1. Valorizar criticamente os resultados da investigação sobre a transmissão de informação aos pacientes e as suas implicações na prática clínica. | 7,9 | 8,0 | 2,0 | 11,4 |
| 1. Descrever os princípios básicos para informar sobre riscos de forma adequada (evitar as manipulações de qualquer tipo e/ou a parcialidade na apresentação de cifras e probabilidades…). | 8,3 | 9,0 | 1,0 | 6,8 |
| 1. Comunicar o risco ao paciente fazendo um uso personalizado dos indicadores (medidas de risco). | 7,7 | 8,0 | 2,0 | 18,2 |
| 1. Suplementar esta informação verbal com diagramas, modelos, informação escrita e instruções quando for necessário. | 8,1 | 8,5 | 1,0 | 11,4 |
| 1. Estimar o nível de conhecimento do paciente sobre o seu problema e até onde deseja saber, para entregar a informação que o paciente realmente requer. | 8,5 | 9,0 | 1,0 | 2,3 |
| 1. Oferecer a informação ao paciente de forma oportuna (circunstância adequada). | 8,3 | 9,0 | 1,0 | 6,8 |
| 1. Adaptar a comunicação ao nível da compreensão e linguagem do paciente, evitando terminologia médica. | 8,7 | 9,0 | 0,0 | 0,0 |
| 1. Proporcionar informação centrada no paciente, incorporando a sua perspetiva e tornando-a significativa para ele. | 8,4 | 9,0 | 1,0 | 4,6 |
| 1. Discutir de maneira centrada no paciente, benefícios, riscos e resultados esperados. | 8,1 | 9,0 | 1,0 | 9,1 |
| 1. Comprovar que o paciente entendeu a informação fornecida, facilitando a expressão de dúvidas. | 8,6 | 9,0 | 0,0 | 2,3 |
| 1. Explicar ao paciente a informação de forma precisa para minimizar a incerteza no processo da tomada de decisões. | 8,1 | 9,0 | 1,0 | 6,8 |
| 1. Partilhar, com o consentimento do paciente, a informação com terceiros(colegas, família e outros…). | 8,0 | 9,0 | 2,0 | 15,9 |

| **A.2.3. Chegar a acordo e Ajudar o paciente a levar a cabo o combinado na tomada de decisões** (O estudante toma decisões considerando a participação e a responsabilidade do paciente, tomando em consideração as suas preferências)  **O estudante será capaz de…** | **X*** | **Me*** | **RI*** | **% fuera Me*** |
| --- | --- | --- | --- | --- |
| 1. Diferenciar os distintos modelos de participação do paciente na tomada de decisões (paternalista, consumista, colaborativa…). | 7,3 | 8,0 | 2,5 | 25,0 |
| 1. Determinar qual deve ser o seu papel mais oportuno como médico no processo de tomada de decisões perante cada paciente. | 7,8 | 8,0 | 2,0 | 13,6 |
| 1. Aceitar o papel da incerteza como elemento substancial do raciocínio clínico e da tomada de decisões. | 7,9 | 9,0 | 2,0 | 13,6 |
| 1. Reconhecer os elementos que contribuem para a presença de incerteza (falta de conhecimentos do profissional, ausência de evidência…) no âmbito da tomada de decisões clínicas. | 7,6 | 8,5 | 2,0 | 20,5 |
| 1. Comunicar aos pacientes que existe incerteza de maneira adaptada ao grau de tolerância deste. | 7,8 | 8,0 | 2,0 | 22,7 |
| 1. Explorar as necessidades, os recursos (informação, autonomia, confiança, responsabilidade, traços psicológicos…) e disposição do paciente para facilitar a sua implicação na tomada de decisões. | 8,3 | 9,0 | 1,0 | 4,6 |
| 1. Alcançar acordos com o paciente utilizando habilidades de negociação. | 8,0 | 9,0 | 1,5 | 6,8 |
| 1. Compreender o papel na prática clínica das ajudas para tomada de decisões (*decision aids*). | 6,9 | 7,5 | 2,0 | 29,6 |
| 1. Capacitar o paciente na utilização de ajudas para a tomada de decisões para utilizá-las na discussão. | 6,9 | 8,0 | 3,0 | 29,6 |
| 1. Esclarecer com o paciente como e quando tem de tomar a decisão. | 7,7 | 8,0 | 2,0 | 15,9 |
| 1. Discutir com o paciente o espetro das possíveis consequências de uma decisão (explicar-lhe as consequências de escolher/não escolher a opção discutida). | 8,0 | 8,0 | 1,0 | 6,8 |
| 1. Oferecer ao paciente a opção de abrir e enriquecer a discussão de tomada de decisões incluindo terceiros (colegas, familiares). | 7,9 | 8,0 | 2,0 | 11,4 |
| 1. Usar o consentimento informado de forma que o paciente compreenda as características e as consequências do procedimento. | 8,3 | 9,0 | 1,0 | 6,8 |
| 1. Adaptar o plano/intervenção aos recursos e às forças do paciente. | 8,5 | 9,0 | 1,0 | 0,0 |
| 1. Fechar o processo no final da consulta utilizando as estratégias comunicacionais adequadas (resumindo, ressaltando aspetos chaves, antecipando possíveis evoluções e orientando na relação…). | 8,7 | 9,0 | 0,5 | 0,0 |
| 1. Assumir o envolvimento do paciente e da sua responsabilidade no processo da tomada de decisões e empregar condutas adequadas para isso. | 7,8 | 8,0 | 2,0 | 20,5 |
| 1. Estar disposto a reavaliar e rever as suas próprias decisões. | 8,2 | 9,0 | 1,0 | 9,1 |

| 1. **COMUNICAÇÃO COM A FAMÍLIA DO PACIENTE**   **B.1. Contexto familiar do paciente** (O estudante reconhece e avalia o papel da família na atenção clínica a pacientes e estabelece uma comunicação efetiva com ela em benefício deste)  **O estudante será capaz de…** | **X** | **Me** | **RI** | **% fuera Me** |
| --- | --- | --- | --- | --- |
| 1. Conhecer o papel da família no sistema na atenção e cuidados dos pacientes. | 8,2 | 9,0 | 1,5 | 9,1 |
| 1. Descrever os modelos básicos que explicam o comportamento das famílias e do paciente como um dos seus membros (o ciclo vital individual e familiar). | 7,6 | 8,0 | 2,0 | 20,5 |
| 1. Conhecer e empregar os métodos e as ferramentas para a identificação da estrutura e o funcionamento familiar (genogramas, ou familiogramas, função familiar, acontecimentos stressantes, rede social…). | 7,2 | 7,0 | 3,0 | 27,3 |
| 1. Considerar os padrões de resposta da família perante a doença e os acontecimentos vitais stressantes quando se atende a um paciente. | 7,6 | 8,0 | 2,0 | 15,9 |
| 1. Identificar o(s) membro(s) da família que cumpre(m) o papel de cuidador principal, para o(s) incorporar no processo e valorizá-lo(s) neste papel. | 7,9 | 9,0 | 1,0 | 11,4 |
| 1. Solicitar e sintetizar a informação relevante de outros membros da família e cuidadores do paciente, se for necessário e se encontrem disponíveis. | 8,0 | 8,0 | 1,5 | 9,1 |
| 1. Estabelecer uma comunicação efetiva com o paciente e a sua família para a identificação dos problemas, deteção dos recursos e a entrada em funcionamento dos planos de atuação em benefício do paciente. | 8,4 | 9,0 | 1,0 | 2,3 |
| 1. Ajudar a família a tomar decisões quando o paciente é menor de idade ou se encontra incapacitado (demências, pacientes em coma, problemas mentais incapacitantes…). | 8,5 | 9,0 | 1,0 | 2,3 |
| 1. Reconhecer desafios comunicacionais específicos com familiares (a confidencialidade, o secretismo, o acompanhante doente…). | 8,4 | 9,0 | 1,0 | 0,0 |
| 1. Mostrar disposição para incorporar a família e trabalhar com ela em benefício do paciente. | 8,3 | 9,0 | 1,5 | 4,6 |
| 1. Mostrar disposição para facilitar a comunicação entre os membros da família do paciente utilizando condutas adequadas para isso. | 7,6 | 8,5 | 2,0 | 13,6 |
| 1. Mostrar sensibilidade com os medos e as preocupações dos familiares utilizando condutas adequadas para isso. | 8,3 | 9,0 | 1,0 | 2,3 |

| 1. **COMUNICAÇÃO INTRAPESSOAL (AUTO PERCEÇÃO)**   **C.1. O médico como pessoa (autoconhecimento, autorreflexão, autocrítica y autocuidados)** (O estudante habitualmente reflexiona sobre o seu comportamento e a forma pela qual comunica, desenvolvendo e melhorando o seu autoconhecimento, autorreflexão, autocrítica, autocuidados)  **O estudante será capaz de…** | **X** | **Me** | **RI** | **% fuera Me** |
| --- | --- | --- | --- | --- |
| 1. Descrever os fatores que influenciam na relação entre o profissional e o paciente (estereótipos, preconceitos socioculturais, experiências, interesses…). | 7,9 | 8,5 | 2,0 | 15,9 |
| 1. Refletir criticamente sobre o seu próprio estilo comunicativo e comportamental considerando as possíveis alternativas. | 8,3 | 9,0 | 1,0 | 4,6 |
| 1. Reconhecer as barreiras que dificultam o autoconhecimento, e utilizar técnicas e estratégias para a sua promoção (prática reflexiva, perspetiva pessoal…). | 8,3 | 9,0 | 1,0 | 6,8 |
| 1. Identificar os sinais de sobrecarga de trabalho e stress (insónia, ansiedade, alterações do sono…). | 8,3 | 9,0 | 1,0 | 4,6 |
| 1. Distinguir as principais fontes de erros médicos (deficiente informação ou avaliação das necessidades do paciente, comunicação inadequada…). | 8,1 | 9,0 | 1,5 | 4,6 |
| 1. Reconhecer os erros técnicos, sesgos cognitivos e reações emocionais que dificultam o desenvolvimento de relações terapêuticas. | 8,2 | 9,0 | 1,0 | 4,6 |
| 1. Utilizar estratégias para reduzir o stress e a sobrecarga (relaxação, grupos de reflexão, grupos Balint, supervisão e apoio…). | 7,8 | 9,0 | 2,0 | 18,2 |
| 1. Controlar as próprias reações emocionais e trabalhar de forma eficiente, ainda que em situações difíceis (alto grau de sofrimento do paciente, paciente que reclama…). | 8,0 | 9,0 | 1,0 | 11,4 |
| 1. Desenvolver as rotinas mentais necessários para o reconhecimento de sesgos próprios, mediante a utilização de técnicas específicas (perguntas reflexivas, observação preconceituosa…). | 8,0 | 8,0 | 2,0 | 6,8 |
| 1. Reconhecer erros próprios (e alheios), assumi-los como parte constitutiva do trabalho e procurar soluções aos mesmos (assistência de superiores…). | 8,5 | 9,0 | 1,0 | 0,0 |
| 1. Reconhecer as suas próprias emoções (insegurança, simpatia/antipatia, atração…) em relação aos outros (pacientes, colegas). | 8,1 | 9,0 | 1,0 | 6,8 |
| 1. Valorizar as forças e as fraquezas pessoais determinantes da sua Autopercepção nos contextos docentes adequados (tutorias, grupos de reflexão…). | 8,2 | 8,5 | 1,0 | 6,8 |
| 1. Aceitar e abordar a incerteza própria de maneira adequada ao estado educativo. | 7,7 | 8,0 | 2,0 | 13,6 |

| 1. **COMUNICAÇÃO INTER-INTRA PROFISSIONAL**   **D.1. O contexto profissional do médico: Comunicação inter e intra profissional** (O estudante comunica eficientemente com os profissionais que fazem parte da sua equipa e fora desta)  **O estudante será capaz de…** | **X** | **Me** | **RI** | **% fuera Me** |
| --- | --- | --- | --- | --- |
| 1. Identificar os princípios básicos da dinâmica de grupo, assim como dos seus fatores favorecedores e inibidores. | 7,7 | 8,0 | 2,0 | 15,9 |
| 1. Identificar os diferentes membros das distintas equipas de saúde interprofissionais e as suas respectivas responsabilidades. | 8,0 | 9,0 | 1,0 | 9,1 |
| 1. Esclarecer o seu próprio papel e responsabilidades como estudante nas equipas profissionais com as quais interatue. | 8,1 | 9,0 | 1,0 | 11,4 |
| 1. Identificar quando referenciar o paciente a profissionais/instituições/agências que possam ajudar a resolver os problemas em função de cada situação. | 8,2 | 9,0 | 1,5 | 6,8 |
| 1. Descrever os princípios e as estratégias para negociar e resolver conflitos com outros profissionais e usá-los adequadamente. | 7,5 | 8,0 | 2,0 | 22,7 |
| 1. Discutir decisões de forma apropriada com colegas, pacientes e os seus familiares, e se for necessário, reavaliar as suas próprias decisões. | 8,2 | 9,0 | 1,0 | 9,1 |
| 1. Assegurar que toda a informação clínica relevante do paciente está disponível. | 7,4 | 8,5 | 2,0 | 18,2 |
| 1. Facilitar o fluxo de informação das opiniões no grupo e promover opiniões divergentes entre os membros da equipa. | 7,9 | 9,0 | 2,0 | 13,6 |
| 1. Dar feedback aos membros da equipa de forma apropriada (comentários em primeira pessoa, ressaltar primeiro o positivo, não julgar). | 8,0 | 9,0 | 2,0 | 9,1 |
| 1. Contribuir de maneira efetiva para a continuidade da atenção na referência/derivação e retorno dos pacientes entre os diferentes níveis assistenciais (primária, especializada). | 7,8 | 8,5 | 2,0 | 13,6 |
| 1. Fazer apresentações clínicas ou científicas em público de forma efetiva. | 7,6 | 8,0 | 2,0 | 15,9 |
| 1. Dar instruções de forma clara e precisa. | 8,3 | 9,0 | 1,0 | 9,1 |
| 1. Contribuir para criar uma atmosfera de trabalho positiva (apoiar e integrar os diferentes membros da equipa, mencionar o lado positivo dos aspetos desagradáveis, valorizar o êxito da equipa…). | 8,4 | 9,0 | 1,0 | 6,8 |
| 1. Respeitar a individualidade, a perceção subjetiva dos membros da equipa e a mestria (perícia) dos diferentes profissionais de saúde. | 8,4 | 9,0 | 1,0 | 6,8 |
| 1. Manter a confidencialidade sobre as decisões tomadas na equipa. | 8,5 | 9,0 | 1,0 | 6,8 |
| 1. Ser assertivo com o resto dos membros da equipa. | 7,8 | 8,0 | 2,0 | 13,6 |
| 1. Mostrar atitude negociadora para alcançar acordos empregando condutas adequadas para isso. | 8,2 | 8,0 | 2,0 | 4,6 |
| 1. Mostrar flexibilidade para mudar o seu papel dentro de uma equipa de trabalho. | 8,3 | 9,0 | 1,0 | 4,6 |

| 1. **COMUNICAÇÃO POR DIFERENTES VIAS**   **E.1. Canais comunicacionais** (O estudante utiliza com eficiência diferentes formas de se comunicar) | | | | |
| --- | --- | --- | --- | --- |
| **E.1.1. Comunicação direta (cara a cara)**  **O estudante será capaz de…** | **X** | **Me** | **RI** | **% fuera Me** |
| 1. Identificar os fatores ambientais (físicos e sociais) que possam obstaculizar a comunicação interpessoal nos diferentes contextos. | 8,3 | 9,0 | 1,0 | 4,5 |
| 1. Identificar se existe discrepância entre os componentes verbais e não-verbais da comunicação. | 8,2 | 9,0 | 1,5 | 9,1 |
| 1. Empregar adequadamente a proxémica (distância física de comunicação). | 8,2 | 9,0 | 1,0 | 6,8 |
| **E.1.2. Comunicação escrita**  **O estudante será capaz de…** | **X** | **Me** | **RI** | **% fuera Me** |
| 1. Reconhecer os formatos e os suportes da história clínica e os documentos habitualmente utilizados para a comunicação escrita com pacientes e entre profissionais (relatórios de alta, derivação, para solicitude de provas,…). | 8,1 | 9,0 | 1,5 | 13,6 |
| 1. Registar a valorização inicial de um paciente assim como a posterior evolução clínica diária numa linguagem escrita concisa e clara. | 8,1 | 9,0 | 1,0 | 13,6 |
| 1. Escrever um relatório de alta e de referenciação, de forma estruturada, compreensível, suficiente e clara. | 8,2 | 9,0 | 1,0 | 11,4 |
| 1. Escrever pedidos de provas e prescrições de forma precisa, clara e justificada. | 8,0 | 9,0 | 2,0 | 11,4 |
| 1. Manter registos claros, apropriados, sobre a informação relevante do encontro clínico. | 8,2 | 9,0 | 1,0 | 9,1 |
| 1. Escrever documentos legais habituais (certidões de óbito, certificados de saúde…). | 8,3 | 9,0 | 1,0 | 6,8 |
| **E.1.3. Comunicação informática o computacional**  **El estudante será capaz de…** | **X** | **Me** | **RI** | **% fuera Me** |
| 1. Reconhecer as diferentes tecnologias da informação mais utilizadas no âmbito da saúde. | 7,7 | 8,0 | 2,0 | 15,9 |
| 1. Conhecer os registos eletrónicos dos pacientes assim como com os sistemas de prescrição e derivação/referenciação. | 8,0 | 8,0 | 2,0 | 6,8 |
| 1. Manipular as tecnologias da informação (e-mails, WhatsApp, web2.0,…) em aspetos de atenção sanitária, garantindo a confidencialidade. | 7,9 | 8,0 | 2,0 | 15,9 |
| **E.1.4. Comunicação telefónica**  **O estudante será capaz de…** | **X** | **Me** | **RI** | **% fuera Me** |
| 1. Reconhecer as utilizações e as limitações da comunicação telefónica com pacientes. | 8,0 | 9,0 | 2,0 | 11,4 |
| 1. Comunicar telefonicamente com pacientes atendendo às procuras específicas e adaptações comunicacionais que este meio requer. | 7,8 | 8,0 | 2,0 | 13,6 |

| 1. **COMUNICAÇÃO EM SITUAÇÕES ESPECIAIS**   **F.1. Contextos comunicacionais específicos** (O estudante aplica e adapta as habilidades comunicacionais nucleares perante situações clínicas específicas e utiliza capacidades concretas que cada situação possa requerer) | | | | |
| --- | --- | --- | --- | --- |
| **F.1.1. Situações sensíveis**  **O estudante será capaz de…** | **X** | **Me** | **RI** | **% fuera Me** |
| 1. Reconhecer situações delicadas que representem desafios comunicacionais (dar más notícias, abordar temas sobre o final da vida, situações de luto, história sexual, violência de género, maltrato infantil, infeção VIH, explicar situações de incerteza clínica…). | 8,5 | 9,0 | 1,0 | 4,5 |
| 1. Abordar algumas delas de maneira sensível e construtiva mediante a aplicação e a adaptação das aptidões comunicacionais nucleares e a utilização de estratégias e habilidades específicas que cada uma delas possa requerer. | 8,2 | 9,0 | 1,0 | 9,1 |
| 1. Conhecer os aspetos legais imprescindíveis vigentes em cada legislação relacionados com a gestão de algumas destas situações. | 7,9 | 9,0 | 2,0 | 13,6 |
| **F.1.2. Manejo das emoções**  **O estudante será capaz de…** | **X** | **Me** | **RI** | **% fuera Me** |
| 1. Reconhecer situações de tensão emocional nas consultas (do tipo stress, temor, irritação, agressividade, negação, colusão, vergonha…). | 8,3 | 9,0 | 1,0 | 4,5 |
| 1. Abordar algumas delas de maneira sensível e construtiva mediante a aplicação e a adaptação das aptidões comunicacionais nucleares e a utilização de estratégias e habilidades específicas que cada uma delas possa requerer. | 7,9 | 8,5 | 1,5 | 9,1 |
| **F.1.3. Diversidade cultural e social**  **O estudante será capaz de…** | **X** | **Me** | **RI** | **% fuera Me** |
| 1. Reconhecer a diversidade cultural e social dos pacientes (etnicidade, nacionalidade, status socioeconómico, idioma, religião, género, valores, sexualidade…) e as dificuldades comunicacionais que isto implica. | 8,3 | 9,0 | 1,0 | 4,5 |
| 1. Abordar algumas delas de maneira sensível e construtiva mediante a aplicação e a adaptação das habilidades comunicacionais nucleares e a utilização de estratégias e habilidades específicas que cada uma delas possa requerer. | 7,9 | 8,5 | 1,5 | 9,1 |
| **F.1.4. Promoção da saúde e modificação de condutas**  **O estudante será capaz de…** | **X** | **Me** | **RI** | **% fuera Me** |
| 1. Descrever os princípios básicos da motivação. | 7,7 | 8,5 | 2,0 | 13,6 |
| 1. Reconhecer os estados do processo de mudança no qual se encontra um paciente no momento de modificar condutas ou de seguir tratamentos. | 8,0 | 8,5 | 2,0 | 9,1 |

| 1. Explorar o grau de motivação do paciente para realizar mudanças. | 8,2 | 9,0 | 1,5 | 6,8 |
| --- | --- | --- | --- | --- |
| 1. Aplicar estratégias comunicacionais eficazes para a modificação de condutas. | 8,1 | 9,0 | 1,0 | 11,4 |
| 1. Assumir um enfoque preventivo e de promoção da saúde na atenção a pacientes e utilizar condutas adequadas para isso. | 8,4 | 9,0 | 1,0 | 2,3 |
| **F.1.5. Contextos clínicos específicos**  **O estudante será capaz de…** | **X** | **Me** | **RI** | **% fuera Me** |
| 1. Abordar alguns contextos clínicos específicos (psiquiátricos, pacientes com demência, com problemas sensoriais: auditivos, visuais, de expressão verbal) mediante a aplicação e a adaptação das aptidões comunicacionais nucleares e a utilização de estratégias e habilidades específicas que cada uma delas possa requerer. | 8,1 | 9,0 | 2,0 | 11,4 |
| **F.1.6. Pacientes de diferentes idades**  **O estudante será capaz de…** | **X** | **Me** | **RI** | **% fuera Me** |
| 1. Comunicar com pacientes de diferentes grupos etários (crianças e pais, adolescentes, idosos) mediante a aplicação e a adaptação das aptidões comunicacionais nucleares e a utilização de estratégias e habilidades específicas que cada um deles possa requerer. | 8,3 | 9,0 | 1,5 | 2,3 |

***X:** Media

***Me:** Mediana

***RI:** Rango intercuartílico

***% fuera de Me:** Porcentaje de encuestados fuera de la región que incluye la mediana
